# Mesocorticolimbic system reactivity to alcohol use-related visual cues as a function of alcohol sensitivity phenotype: A pilot fMRI study

**DOI:** 10.1016/j.addicn.2024.100156

**Published:** 2024-05-07

**Authors:** Roberto U. Cofresí, Spencer Upton, Alexander A. Brown, Thomas M. Piasecki, Bruce D. Bartholow, Brett Froeliger

**Affiliations:** a Department of Psychological Sciences, University of Missouri - Columbia, USA; b Center for Tobacco Research and Intervention and Department of Medicine, University of Wisconsin - Madison, USA; c Department of Psychological and Brain Sciences, University of Iowa, USA; d Department of Psychiatry, University of Missouri - Columbia, USA

**Keywords:** Alcohol, Incentive, Fronto-striatal, Sensitization, Level of response

## Abstract

Low sensitivity (LS) to alcohol is a risk factor for alcohol use disorder (AUD). Compared to peers with high sensitivity (HS), LS individuals drink more, report more problems, and exhibit potentiated alcohol cue reactivity (ACR). Heightened ACR suggests LS confers AUD risk via incentive sensitization, which is thought to take place in the mesocorticolimbic system. This study examined neural ACR in LS and HS individuals. Young adults (*N* = 32, *M*_age_=20.3) were recruited based on the Alcohol Sensitivity Questionnaire (HS: *n* = 16; LS: *n* = 16; 9 females/group). Participants completed an event-related fMRI ACR task. Group LS had higher ACR in left ventrolateral prefrontal cortex than group HS. In group LS, ACR in left caudomedial orbitofrontal cortex or left putamen was low at low alcohol use levels and high at heavier or more problematic alcohol use levels, whereas the opposite was true in group HS. Alcohol use level also was associated with the level of ACR in left substantia nigra among males in group LS. Taken together, results suggest elevated mesocorticolimbic ACR among LS individuals, especially those using alcohol at hazardous levels. Future studies with larger samples are warranted to determine the neurobiological loci underlying LS-based amplified ACR and AUD risk.

## 1. Introduction

People differ dramatically from one another in the extent of their subjective, cognitive, physiological, and behavioral responses to alcohol consumption [1-3]. This inter-individual variability reflects largely heritable, trait-like variation in pharmacodynamic response to acute alcohol [4-6], and moderates risk for alcohol use disorder (AUD). Specifically, lower sensitivity (LS) to acute alcohol—especially to its sedative-like effects—is associated with higher rates of alcohol use-related problems, including AUD [7-9], and may be an endophenotype for AUD risk [10]. Despite considerable evidence supporting LS as a reliable, trait-like indicator of risk for AUD onset or progression, the specific neurobehavioral mechanisms by which LS confers risk for AUD remain poorly understood.

Incentive sensitization may be one mechanism by which LS confers risk for AUD. According to the incentive sensitization theory of addiction (ISTA) [11-14], in vulnerable individuals chronic drug use sensitizes the incentive salience (affective-motivational significance) of the drug and cues associated with its use (for alcohol-specific translational evidence, see: [15,16]). This incentive sensitization manifests as heightened cue-elicited attentional biases, behavioral approach, and subjective craving. In keeping, among individuals with LS compared to peers with higher sensitivity (HS), alcohol-related cues: (i) spontaneously capture visual attention [17,18]; (ii) elicit stronger event-related potential (ERP) responses indicative of attention to cues due to their affective-motivational significance [19-23]; (iii) activate a behavioral disposition to approach to the cue (e.g., promoting physical proximity to the alcohol beverage container) [24,25]; and (iv) elicit greater levels of subjective craving for alcohol in the laboratory [21] and the natural environment ([26], but see: [27]).

As it applies to rewards and reward-related cues, the construct of incentive salience has been tied to activity in the mesocorticolimbic system. Rewards and reward-predictive cues alike activate the mesocorticolimbic system in rodents [28-30], and that activation scales with behavioral indices of incentive salience attribution [31-35]. Additionally, in rodents, the behavioral propensity to attribute more incentive salience to reward-predictive cues is linked to drug cue reactivity in various models of relapse to drug seeking, and demonstrated for several different drug classes [36-41]. Additionally, functional magnetic resonance imaging (fMRI) studies in humans have shown that both rewards and reward-related cues activate the mesocorticolimbic system [28,42,43]. In particular, fMRI studies have shown that the mesocorticolimbic system is hyper-reactive to substance use-related cues among individuals with a substance use disorder [44-49], including those with AUD [50,51].

We conducted a pilot fMRI study to examine LS vs. HS group differences in mesocorticolimbic ACR among young adults who regularly use alcohol. Given the proposal that LS to alcohol confers risk to AUD *via* incentive sensitization, we hypothesized that ACR in the mesocorticolimbic system would be greater among LS compared to HS individuals. Although the study was focused on providing insight into the neurobiological loci underlying alcohol sensitivity-based differences in differential reactivity to alcohol compared to control cues, food/drink and nicotine cues were included in the fMRI cue reactivity task to allow examination of the *specificity* of alcohol sensitivity phenotype-based differences in reward cue reactivity.

## 2. Methods

### 2.1. Participants

Participants were recruited between 11/2021 and 04/2022 from a pool of individuals involved in an NIH-funded longitudinal study (AA025451) characterizing alcohol sensitivity across early emerging adulthood. Inclusion criteria for the parent study were: age 18 to 20 years at enrollment, ability to read and write English, normal or corrected-to-normal visual acuity, and regular alcohol use (at least monthly alcohol use in the past year and at least 1 binge-drinking episode in the past 6 months). Exclusion criteria for the parent study included a history of unsuccessful attempts to quit or moderate alcohol use and considerations related to EEG recording (for more details, see [23]). Exclusion criteria for the current study included: no longer residing in the community (Columbia, MO); moderate alcohol sensitivity phenotype (see 2.3.1 Self-Reported Alcohol Use and Alcohol Sensitivity for details), current or past psychosis (including manic episodes) as evidenced by current or past use of certain medications (e.g., haloperidol, lamotrigine, lithium, risperidone); history of neurological disease (e.g., epilepsy); history of prior head injuries that resulted in a loss of consciousness; non-removable electronics or metal; anxiety or claustrophobia in the MR scanner bore; body size that prevents comfortable fit in the MR scanner bore; and for female participants: currently pregnant, nursing, or trying to become pregnant. There were no inclusion/exclusion criteria surrounding the use of substances other than alcohol. Of 93 individuals who completed the combined eligibility screening survey and baseline assessment for the current study, 76 were eligible. Eligible individuals were enrolled in a way that ensured similar numbers of males and females, and similar numbers of individuals endorsing current use of any nicotine products (see Supplemental Information for assessment details), in two groups reporting either extreme HS or LS to alcohol in daily life (see 2.3.1 Self-Reported Alcohol Use and Alcohol Sensitivity for details on how groups were defined; see Table S1 for details on nicotine use by group). One HS individual was removed from the analysis for not completing the MRI portion of the laboratory visit. The final sample size for the current study thus consisted of 32 individuals: 16 HS and 16 LS. Sample socio-demographics are presented in Table 1 and were tested for equivalence between groups using either X^2^ or non-directional Mann-Whitney-Wilcoxon *U tests*, as appropriate.

### 2.2. Procedures

Laboratory visits (2 hr duration) took place at the University of Missouri Cognitive Neuroscience Systems Core facility. Visits took place Monday through Friday, and 30 out of 32 visits started between 12pm and 4pm (two visits started before noon). To equate hunger levels, participants were asked to fast for 2 hrs before arrival (verified at arrival *via* self-report) and were served a light snack (granola bar) with water at arrival. Breath alcohol samples were taken to verify sobriety. Once informed consent was obtained, questionnaires were administered, including past-week craving measures, and then participants underwent training in a mock MRI scanner to acclimate them to the MRI environment. Urine samples were obtained from all participants and tested for cotinine (Healgen One Step COT, Healgen Scientific LLC, Houston, TX, USA). Urine samples from female participants also were tested for pregnancy (Sure-Vue hCG-STAT, Fisher Healthcare, Pittsburgh, PA, USA). All participants then underwent the MRI phase of the study. Following the acquisition of a T1 anatomical scan, a B0 field map was acquired. Functional images were then acquired during *a* ≈ 7 min response inhibition task (not reported here). A second B0 field map was then acquired. Participants then completed pre-task momentary craving self-report. Next, functional images were acquired while participants completed a cue reactivity task (6 runs; ≈4 min/run; see 2.4.3 Alcohol Cue Reactivity fMRI Task for details). Participants were allowed to rest for ≈1 min between task runs. After completion of the cue reactivity task (≈30 min total), participants completed post-task momentary craving self-report and then were removed from the scanner. Participants then completed an 8-day TimeLine Follow-Back (TLFB) calendar [52]. Finally, participants were debriefed and compensated (50 USD). All procedures were approved by the University of Missouri Institutional Review Board.

### 2.3. Questionnaires

#### 2.3.1. Self-Reported alcohol use and alcohol sensitivity

Past year alcohol use was assessed using a standardized question set [53]. Sensitivity to the acute effects of alcohol in daily life was assessed using the Alcohol Sensitivity Questionnaire (ASQ), a 15-item retrospective measure that has been validated using a placebo-controlled alcohol challenge in the laboratory [54]. Internal consistency reliability (ICR) for ASQ scores was excellent (*α*=0.91–0.95). Individuals were classified as LS or HS at recruitment for the current study based on whether their ASQ total score was above or below sex-specific thresholds delimiting the upper or lower terciles of the sex-stratified ASQ total score distribution, respectively. Individuals whose ASQ total scores fell in the middle tercile for either sex were not invited to enroll in the current study. These sex-specific thresholds were based on sex-stratified ASQ score distributions obtained from prior independent survey studies of emerging adults (18–25 yr; *N* = 5244, 59 % female) enrolled in our department’s introductory psychology course between fall 2013 and spring 2017. Specifically, for the current study, group LS was comprised of females with ASQ total scores > 4.50, and males with ASQ total scores > 5.50. Group HS was comprised of females with ASQ total scores < 3.00,and males with ASQ total scores < 4.50. The Alcohol Use Disorders Identification Test (AUDIT) also was completed [55,56]. ICR for AUDIT scores was fair-to-excellent (*α*=0.78–0.88). Finally, at the end of the lab visit, participants indicated on an 8-day TLFB calendar on which days they used alcohol and how many standard drinks (14 g ethanol) were consumed. Experimenters then reviewed the TLFB calendar with participants. For every day in the calendar on which alcohol use was indicated, experimenters asked the participant questions about the number of drinking episodes, the time of day for each episode and its duration, as well as questions about whether any "problems" or "negative consequences" resulted from their alcohol use on that day such as whether they had a "hangover" the next day or realized the next day that they had "blacked out" or "had any other problem that they think was related to their alcohol use the previous day."

#### 2.3.2. Self-Reported craving for alcohol

Alcohol cravings were assessed using the Craving Experience Questionnaire (CEQ; [57]), which contains items capturing the frequency (CEQ-F) and strength/intensity (CEQ-S) of cravings in the past week. ICR for alcohol CEQ-F and CEQ-S was good-to-excellent (*α*=0.82–0.95). In the scanner, momentary alcohol craving intensity level was assessed using a single-item measure ("How much do you want to drink alcohol right now?"; response options ranged from "not at all" [1] to "a lot" [8] in 1-unit increments).

### 2.4. MRI

#### 2.4.1. Image acquisition

MRI scans were acquired using a 3T Siemens Prisma scanner using a 32-channel headcoil. A high-resolution, T1-weighted MPRAGE sequence (TR=2300 ms, TE=2.26 ms, flip angle=9°, 192 slices, 1-mm isotropic voxels, FOV=256 mm) was used to acquire anatomical images. Functional T2*-weighted images were acquired to measure BOLD responses using a simultaneous multi-slice (SMS) echo-planar imaging (EPI) sequence (acceleration factor=3, TR=2000 ms, TE=36 ms, flip angle=70°, 69 slices, 2.2-mm isotropic voxels, FOV=207 mm).

#### 2.4.2. Image processing

Functional and structural images underwent standard preprocessing in Matlab version 2021b (The Mathworks Inc., Natick, MA, USA), using statistical parametric mapping (SPM) package version 12 [58]. Pre-processing of functional images included: B0 correction; realignment; slice timing correction; co-registration to structural images; segmentation of structural images; normalization to MNI space using forward deformations with resampling to 1.5-mm^3^ voxels; and smoothing with a 6-mm^3^ full-width at half maximum (FWHM) Gaussian filter.

#### 2.4.3. Alcohol cue reactivity fMRI task

This event-related fMRI task consisted of 6, 4-minute runs. Inside each run, 50 trials were presented. Each trial consisted of a centrally presented ≈3 s white fixation crosshair (jittered: 1–5 s in 0.5 s steps) followed by an image (fixed duration: 2 s) from 1 of 5 cue types (described below), always on a black background. Images were not allowed to repeat across runs to minimize habituation (10 images per cue type per run). The sequence of images within each run was randomized across participants. The visual angle subtended was standardized across images (8.6 ° tall x 6.2 ° wide).

Cue types were: Alcohol (Alc), Food/Drink (F/D), Nicotine (Nic), Complex Control (CC), and Simple Control (SC). Alc cues depicted alcohol beverages (beer, wine, liquor) and/or their use. F/D cues depicted non-drug ingested natural rewards (food, soft-drinks) and/or their ingestion. Nic cues depicted electronic nicotine delivery systems (ENDS) and/or their use. A version of the task in which Nic cues depicted tobacco cigarettes and/or their use also had been prepared, but since no participants endorsed current tobacco cigarette smoking, it was not used. All Alc and F/D cues were taken from previously published picture sets [22,59-62] except for 6 Alc cues and 8 F/D cues, which were obtained from searches for images under Creative Commons licenses within digital stock photography repositories. Nic (ENDS) cues were obtained from similar searches, except for 18 which were obtained from a previously published picture set [63]. CC cues depicted neutral-valence, low-arousal real-world scenes and objects and/or their use, and all were taken from the International Affective Picture Set [64] (see Supplemental Information for details). SC cues were illustrations of simple geometric shapes made by the authors using digital vector graphics software. Example stimuli are shown in Figure S1.

### 2.5. Analytic approach

#### 2.5.1. Behavior

Expected differences in alcohol use and craving as a function of alcohol sensitivity group (i.e., LS>HS) were tested. Mann-Whitney-Wilcoxon *U tests* were used because alcohol use and craving measures are non-normally distributed. The threshold for significance was *p*<.05. No correction for multiple tests was applied.

#### 2.5.2. Alcohol cue reactivity fMRI task

##### 2.5.2.1. First-Level analyses.

Preprocessed functional images were entered into a 1st level analysis using the general linear model (GLM) to examine the blood-oxygen-level dependent (BOLD) response to each of the 5 cue types: Alc, F/D, Nic, CC, and SC. Each cue type was modeled using a delta regressor (event duration=2 s) and convolved with a canonical hemodynamic response function. Intra-run motion was removed through rigid body rotation and translation, and 6 motion parameters were included as nuisance covariates. A high-pass filter (128 s; 0.008 Hz) was applied to remove slow signal drift.

Reward-specific cue reactivity (CR) was defined as the response to Alc, F/D, or Nic *above and beyond* the response to CC (i.e., Alc>CC, F/D>CC, Nic>CC contrasts; henceforth: ACR, FCR, NCR), a typical approach for isolating reactivity to the meaning of, or value associated with, pictorial stimuli depicting rewards from general reactivity to complex visual stimuli [59,65]. ACR and FCR contrast maps were used in the 2nd level analyses described next. NCR contrast maps were analyzed at the 2nd level too, but these are presented in Supplemental Information because <30 % of the sample endorsed nicotine use, limiting the interpretability of NCR.

##### 2.5.2.2. Second-Level analyses: Mesocorticolimbic mask.

To detect ACR or FCR regions within the mesocorticolimbic system in the present sample, a 2nd level model was fit using an explicit mask consisting of 6 cortical (anterior cingulate, frontal gyrus [inferior, medial, middle, superior], and orbital gyrus) and 5 subcortical structures (amygdala, caudate, nucleus accumbens, putamen, and substantia nigra) made in Wake Forest University (WFU) Pickatlas version 3.0.5 [66-68]. This 2nd level analysis was blind to alcohol sensitivity phenotype group and sex, and no nuisance covariates were included. To detect voxel clusters showing significant ACR or FCR across the sample, the voxel intensity-based statistical threshold was set to family-wise error (FWE)-corrected *p*<.05 using the random field theory (RFT) method implemented in SPM, which accounts for image smoothing and the statistical dependency of signal from neighboring voxels. Furthermore, the cluster spatial extent-based statistical threshold was set to kE > 1 to avoid detecting intensely activated but spatially isolated voxels since these are more likely to be false positives.

Person-level contrast beta coefficients were then extracted using MarsBaR version 0.45 [69]. Specifically, the average contrast beta coefficient in a sphere (radius=5-mm) centered on the peak voxel in each cluster was extracted as a measure of ACR or FCR at each empirically determined functional region of interest (ROI). Peak voxel locations are reported using the Montreal Neurological Institute (MNI) coordinate system.

##### 2.5.2.3. Second-Level analyses: exploratory whole-brain.

For completeness, a parallel set of exploratory whole-brain models also were fit and are reported. The method and thresholding were the same as those described above for the masked 2nd level analyses.

##### 2.5.2.4. Second-Level analyses: post-hoc subcortical atlas.

This analysis was conducted following the failure to observe ACR in subcortical nodes in the mesocorticolimbic system masked 2nd level analysis. Left and right hemisphere-specific explicit masks were made for each anatomically defined subcortical ROI (amygdala, nucleus accumbens, caudate, putamen, substantia nigra) using WFU Pickatlas, specifically the IBASPM71 and Talairach Daemon (TD) atlases. The beta coefficient corresponding to the Alc - CC contrast was extracted from every person (1st level models described above), using MarsBaR version 0.45, as the average across all estimated beta coefficients (1 per voxel) within each anatomical ROI mask.

#### 2.5.3. Brain-behavior associations

##### 2.5.3.1. Alcohol sensitivity hypothesis tests.

The predicted alcohol sensitivity phenotype-based group difference (i.e., LS>HS) was tested at all identified ROIs for ACR using a multiple linear regression (MLR) approach. This approach enables testing and controlling for moderating effects of sex and differences in alcohol use, problems, or cravings between groups. Differences in alcohol use (AUDIT Consumption scores), problems (AUDIT Problem scores), and craving (frequency: CEQ-F Intensity scores; intensity: CEQ-S Intensity scores) were tested as moderators of the alcohol sensitivity group difference in separate models. These potential moderator variables were entered into the MLR models as grand mean-centered continuous covariates. Testing potential moderation in separate models was necessary to mitigate collinearity issues arising from large correlations between the examined potential moderators. Sex was tested as a potential moderator in all models using an effect-coded binary variable. A model selection process was used to find the best-fitting MLR model, defined as the most parsimonious model that explains a significant amount of between-person variance. This iterative process began with a 3-way interaction model and involved dropping non-significant interaction effects followed by non-significant main effects. The threshold for significance was *p*<.05. Bonferroni correction was applied to comparisons of simple slopes or model-estimated means conducted to decompose significant interaction effects. MLR model results that are not relevant to the alcohol sensitivity hypothesis are reported in Supplemental Information.

##### 2.5.3.2. Exploratory - EEG-ERP.

Given that participants’ EEG was recorded during an alcohol cue reactivity task at a prior lab visit for the parent study, links could be explored between an EEG-ERP measure of differential incentive salience attribution to alcohol cues and measures of ACR in BOLD at all ROIs in the current study. To the extent both measures index the same construct (i.e., incentive salience attribution to alcohol cues or ACR more broadly), we speculate that the two neural measures may demonstrate a significant association with one another. Similar BOLD-ERP correlations have been found for measures of reward anticipation [70] and reward receipt [71]. Since participants’ scores on the EEG-ERP measure of differential incentive salience attribution to alcohol cues were part of a previous publication [23], confirmatory analyses of ACR in the EEG-ERP measure for the current subsample of participants are presented in Supplemental Information alongside a summary of the EEG-ERP method. To examine the main effects of between-person differences in the EEG-ERP measure on BOLD-ACR as well as potential moderation effects on the alcohol sensitivity group difference in BOLD-ACR, the MLR model testing strategy described above was applied. The threshold for significance was *p*<.05. No correction for multiple tests was applied.

## 3. Results

### 3.1. Behavior

As shown in Table 2, past-year and past-week alcohol use behavior was significantly more frequent and intense in group LS than HS (e.g., more drinking days, more drinks per drinking day, greater AUDIT Consumption scores). Past-year alcohol use-related problems also were significantly higher in group LS than HS (i.e., greater AUDIT Problem scores), although past-week alcohol use-related problems per drinking day were not significantly elevated for group LS than HS. Past week’s alcohol craving was more frequent (greater CEQ-F Intensity scores) and intense (greater CEQ-S Intensity scores) in group LS than HS. However, in the scanner, alcohol craving was similar between groups pre- and post-task, as were within-person craving change scores.

### 3.2. Brain and brain-behavior associations

#### 3.2.1. Second-Level analyses: Mesocorticolimbic mask results

**Fig. 1.**
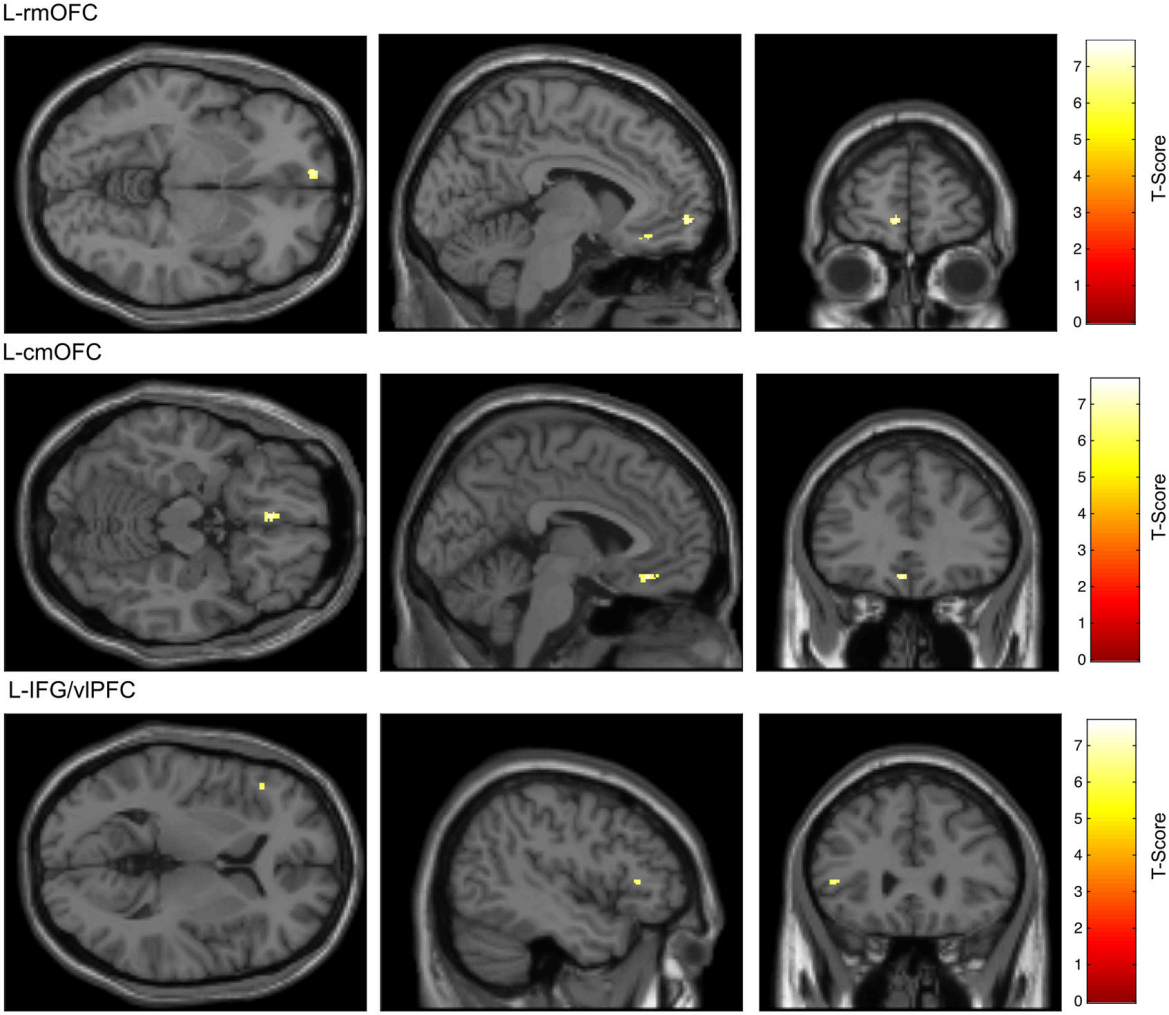
Frontocortical areas that exhibited alcohol cue-specific reactivity (ACR) in the mesocorticolimbic system-masked 2nd-level fMRI BOLD analysis *Note*. ACR was visualized using the alcohol cue (Alc) > affectively neutral complex cue (CC) BOLD contrast (Alc-CC). The intensity and extent of activations in this contrast that survived random field theory (RFT)-based family-wise error (FWE) correction to *p*<.05 are shown on a canonical T1-weighted image of the human brain included with SPM. Data represent *N* = 32 healthy emerging adults reporting regular alcohol use. **Top row**: ACR in the left hemisphere rostral medial orbitofrontal cortex (L-rmOFC). **Middle row**: ACR in the left hemisphere caudal medial orbitofrontal cortex (L-cmOFC). **Bottom row**: ACR in the left hemisphere inferior frontal gyrus pars triangularis, a.k.a., the left hemisphere ventrolateral prefrontal cortex (L-IFG/vlPFC).

As shown in Table 3, three clusters were identified for ACR, all of which were in the frontal cortex. Two of the three clusters were in the left medial orbitofrontal cortex (L-mOFC): one in the caudal inferior aspect of the gyrus rectus (L-cmOFC; Fig. 1), and one in the rostral aspect of the medial orbital gyrus (L-rmOFC; Fig. 1). The third cluster was in the left inferior frontal gyrus pars triangularis (L-IFG), which is also known as the left ventrolateral prefrontal cortex (L-vlPFC; Fig. 1). No clusters were identified for FCR.

##### 3.2.1.1. Alcohol sensitivity hypothesis test.

Significant MLR models were found for between-person variation in ACR in l-cmOFC and l-vlPFC, but not l-rmOFC. The best MLR models of between-person variation in ACR in l-cmOFC {*R*^2^=0.347–0.376, *F*[5, 26]=3.133–2.767, *p*=.024–0.039) indicated the following significant effects relevant to the hypothesis: (i) alcohol sensitivity group x AUDIT Consumption (*p*=.016), and (ii) alcohol sensitivity group x AUDIT Problem (*p*=.005). The best MLR model of between-person variation in ACR in l-vlPFC (*R*^2^=0.204, *F*[3,29]=3.71, *p*=.037) included a significant main effect of alcohol sensitivity group (*p*=.019) and a non-significant main effect of sex (*p*=.263). Below, Bonferroni-corrected comparisons of simple slopes are used to decompose the interaction effects involving alcohol sensitivity group on ACR in l-cmOFC, and comparison of model-estimated means is used to evaluate the alcohol sensitivity group difference on ACR in l-vlPFC.

###### 3.2.1.1.1. ACR in l-cmOFC.

**Fig. 2.**
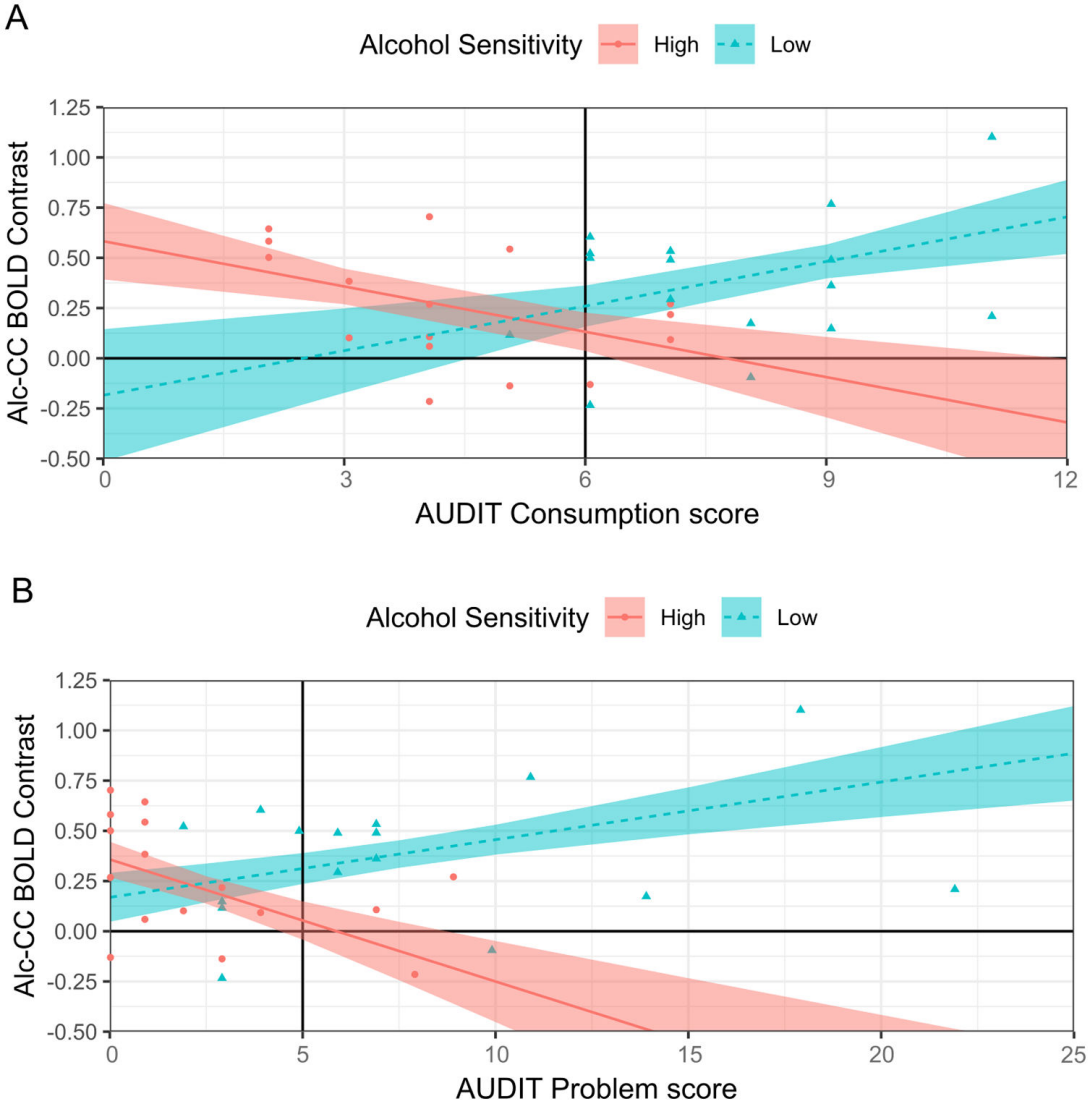
Alcohol sensitivity group x AUDIT subscale interaction on alcohol cue-specific BOLD reactivity in L-cmOFC *Note*. AUDIT=Alcohol Use Disorders Identification Test. Alc=alcohol cues. CC=affectively neutral complex cues. Person-level Alc-CC BOLD contrast beta coefficients belonging to the High (*n* = 16) and Low (*n* = 16) Alcohol Sensitivity groups are shown as red-filled circles and teal-filled triangles, respectively. Multiple linear regression (MLR) model predicted M across levels of each AUDIT subscale are shown in each panel for the High and Low Alcohol Sensitivity groups as a red solid line and dashed teal line, respectively, with the boundaries of the red-filled and teal-filled areas around those lines representing ± 1 SE. AUDIT subscales were entered into the MLR models as grand-mean centered predictors. The grand-mean AUDIT subscale score is shown in each panel as a solid black vertical line intersecting the x-axis.

Decomposition indicated that the alcohol sensitivity group x AUDIT Consumption and alcohol sensitivity group x AUDIT Problem interaction effects were similar, which may reflect the large correlation between AUDIT Consumption and Problem scores (*r* = 0.76, *p* < .001), but also that the sensitivity group x AUDIT Problem interaction effects were stronger. As shown in Fig. 2A-B, the simple slope of AUDIT Consumption differed between group HS and LS (α*b*±SE=0.149±.058, *t*(26)=2.578, *p*=.016), as did the simple slope of AUDIT Problem (Δ*b*±SE=0.089±.029, *t*(26)=3.091, *p*=.005). In group LS, there was a significant, positive simple slope of AUDIT Problem, *b*±SE=0.029±.013, *t*(26)=2.200, *p*=.037, whereas, in group HS, there was a significant, *negative* simple slope of AUDIT Problem, *b*±SE=−0.061±.025, *t*(26)=2.466, *p*=.021. The corresponding simple slopes of AUDIT Consumption trended in the same directions, but were not significant (*p*=.076–0.083).

###### 3.2.1.1.2. ACR in l-vlPFC.

**Fig. 3.**
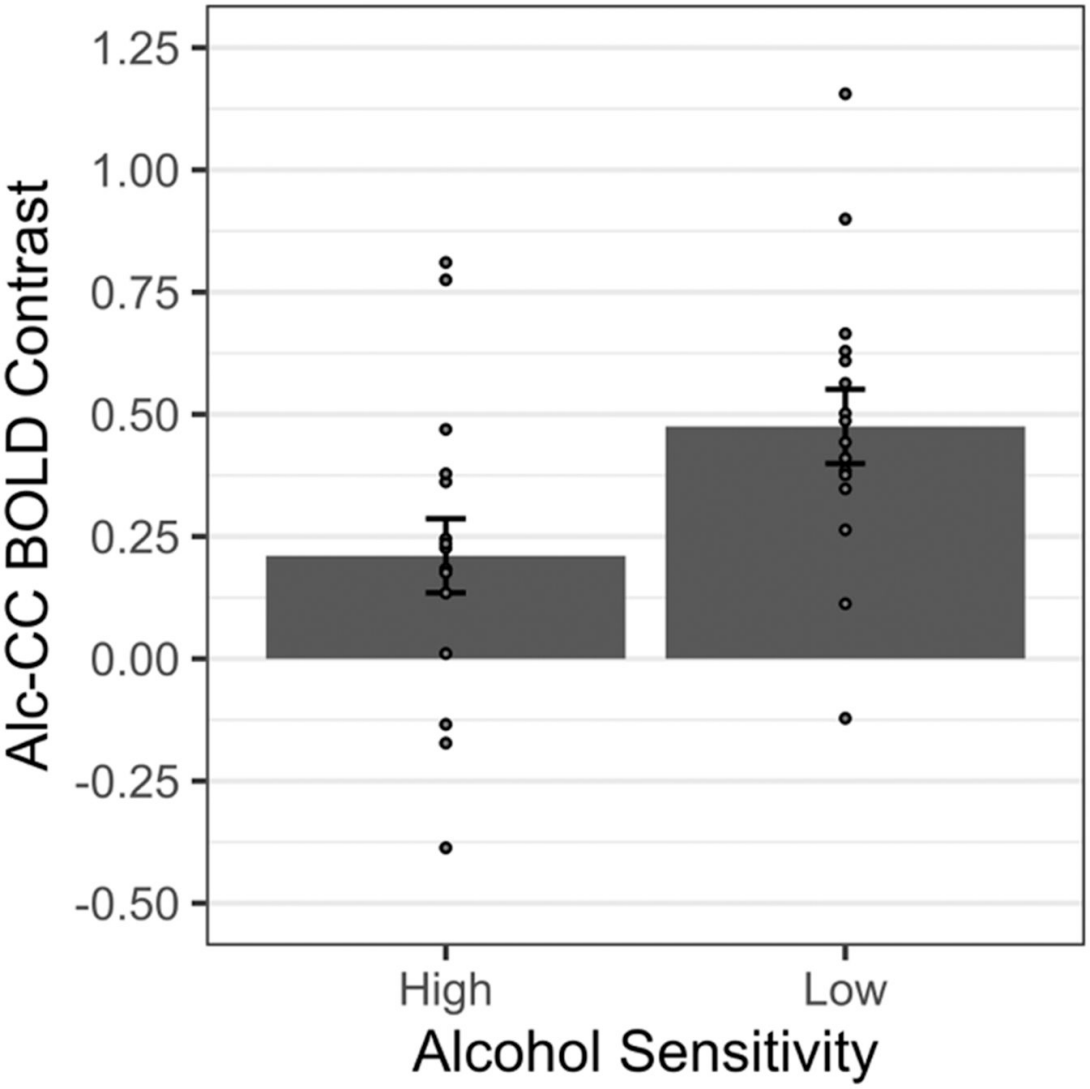
Alcohol sensitivity group effect on alcohol cue-specific BOLD reactivity in l-vlPFC *Note*. Alc=alcohol cues. CC=affectively neutral complex cues. Person-level Alc-CC BOLD contrast beta coefficients are shown as gray-filled circles. Gray bars show the multiple linear regression (MLR) model predicted Alc-CC BOLD contrast beta coefficient *M* ± 1 SE for the groups representing High and Low Alcohol Sensitivity, respectively. *N* = 16/group.

Comparison of model-estimated means (controlling for sex) indicated that ACR was significantly greater in group LS than HS, M_D_±SE_D_=0.264±.107, *t*(29)=2.474, *p*=.019 (see Fig. 3).

#### 3.2.2. Second-Level analyses: exploratory whole-brain results

As shown in Table S2, eight clusters were identified for ACR. There was partial internal replication of ACR clusters identified by mesocorticolimbic mask 2nd level model analysis. Specifically, the ACR clusters in l-rmOFC and l-cmOFC were re-identified, but the one in l-vlPFC was not. Additional ACR clusters were identified in vision-related posterior cortices, in keeping with prior fMRI studies of alcohol and drug cue reactivity [72]. As shown in Figure S2, ACR clusters were located in the left and right hemisphere fusiform gyrus, the left and right hemisphere occipital gyrus (inferior and middle), and the left hemisphere posterior cingulate (dorsal and ventral). No clusters were identified for FCR.

##### 3.2.2.1. Alcohol sensitivity hypothesis test.

No significant MLR models were found for between-person variation in ACR at any of the voxel clusters identified outside the mesocorticolimbic system.

#### 3.2.3. Second-Level analyses: post-hoc subcortical atlas results

##### 3.2.3.1. Alcohol sensitivity hypothesis test.

Significant MLR models were found for between-person variation in ACR across the l-putamen and l- and R-substantia nigra (SN). No significant MLR models were found for between-person variation in ACR across the l- or R- amygdala, l- or R-nucleus accumbens, l- or R- caudate, or R-putamen. The best MLR model of between-person variation in ACR across the l-putamen (*R*^2^=0.348, *F*[4, 27]=3.602, *p*=.018) indicated a significant alcohol sensitivity group x AUDIT Consumption interaction effect (*p*=.016). The best MLR models of between-person variation in ACR across the l-SN (*R*^2^=0.351–0.426, *F*[5–7, 24–26]=2.548–2.819, *p*=.036–0.041) indicated one significant effect relevant to the hypothesis: (i) an alcohol sensitivity group x sex x AUDIT Consumption interaction (*p*=.010). Below, Bonferroni-corrected comparisons of simple slopes are used to decompose the significant interaction effects involving alcohol sensitivity group on ACR across the l-putamen and l-SN.

###### 3.2.3.1.1. ACR in l-putamen.

**Fig. 4.**
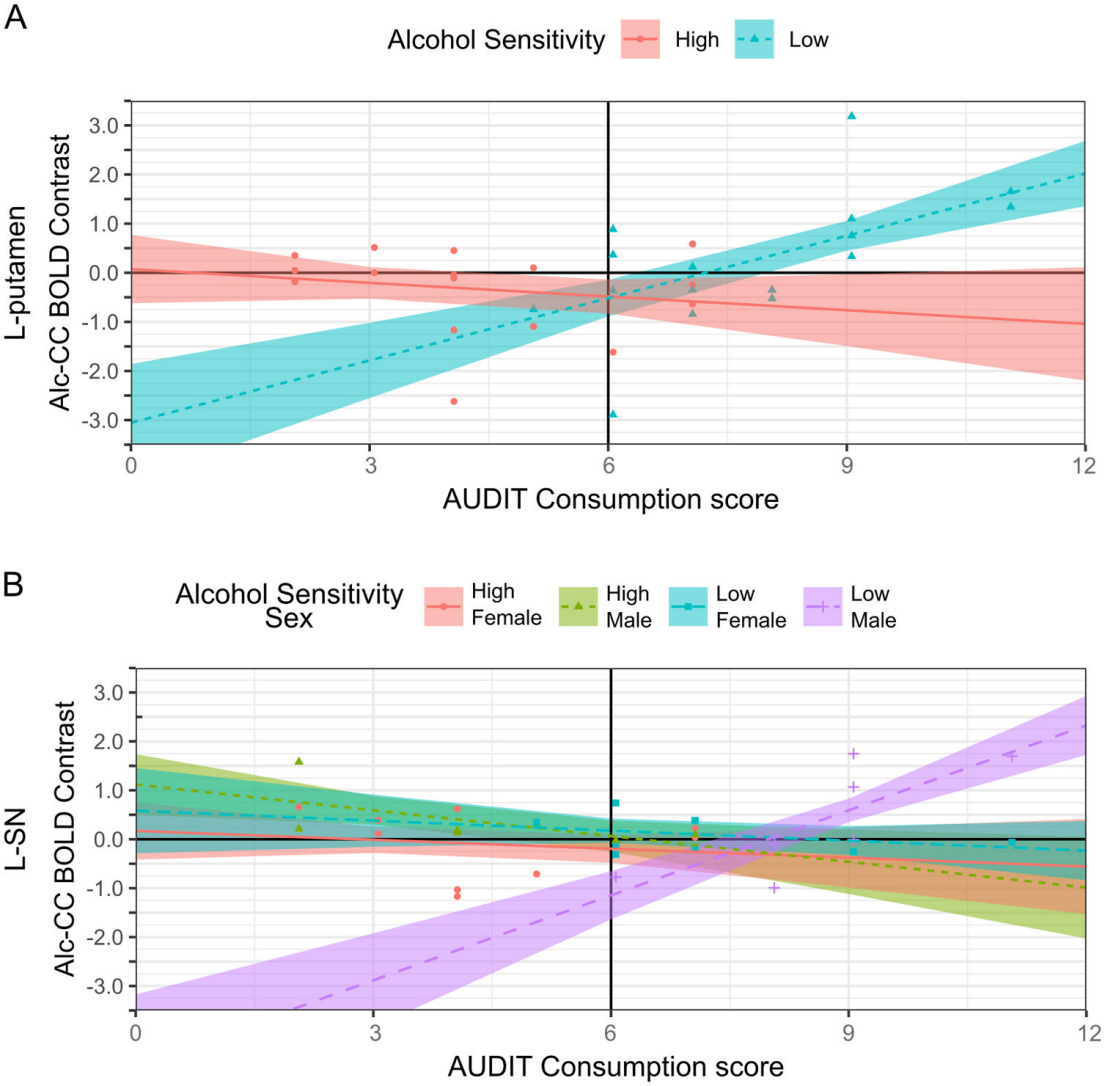
Alcohol sensitivity group x AUDIT Consumption interaction on alcohol cue-specific BOLD reactivity in l-putamen (**A**) and alcohol sensitivity group x sex group x AUDIT Consumption interaction on alcohol cue-specific BOLD reactivity l- substantia nigra (SN; **B**) *Note*. AUDIT=Alcohol Use Disorders Identification Test. Alc=alcohol cues. CC=affectively neutral complex cues. **A:** Person-level l-putamen Alc-CC BOLD contrast beta coefficients belonging to the High (*n* = 16) and Low (*n* = 16) Alcohol Sensitivity groups are shown as red-filled circles and teal-filled triangles, respectively. Multiple linear regression (MLR) model predicted M across levels of the AUDIT Consumption subscale are shown in each panel for the High and Low Alcohol Sensitivity groups as a red solid line and dashed teal line, respectively, with the boundaries of the red-filled and teal-filled areas around those lines representing ± 1 SE. **B:** Person-level l-SN Alc-CC BOLD contrast beta coefficients belonging to High Alcohol Sensitivity Female (*n* = 9), High Alcohol Sensitivity Male (*n* = 7), Low Alcohol Sensitivity Female (*n* = 9), and Low Alcohol Sensitivity Male (*n* = 7) are shown as red-filled circles, green-filled triangles, teal-filled squares, and purple-filled crosshairs, respectively. MLR model predicted M across levels of the AUDIT Consumption subscale are shown for each cell of the alcohol sensitivity group x sex group interaction using red, green, teal, and purple lines of varying types (e.g., solid, dashed, dotted), respectively. The boundaries of the correspondingly colored areas around each line represent ± 1 SE. **A-B**: AUDIT Consumption was entered into the MLR models as a grand-mean centered predictor. The grand-mean AUDIT Consumption score is shown in each panel as a solid black vertical line intersecting the x-axis.

As shown in Fig. 4A, the simple slopes of AUDIT Consumption differed between group HS and LS (Δ*b*±SE=0.516±.210, *t*(27)=2.545, *p*=.021). In group LS, there was a significant, positive simple slope of AUDIT Consumption, *b*>±SE=0.423±.149, *t*(27)=2.840, *p*=.008, whereas in group HS, the simple slope of AUDIT Consumption did not differ significantly from null, *b*±SE=−0.093±.148, *t*(27)=0.628, *p*=.535.

###### 3.2.3.1.2. ACR in l-SN.

As shown in Fig. 4B, the simple slopes of AUDIT Consumption differed by alcohol sensitivity and sex groups. The simple slope of AUDIT Consumption among males in group LS differed significantly from the simple slope of AUDIT Consumption in each other cell of the alcohol sensitivity group x sex interaction, Δ*b*>±SE ≥ 0.640±.207, *t*(24) ≥ 3.083, *p* ≤ 0.030. Specifically, there was a significant, positive simple slope of AUDIT Consumption among males in group LS, *b*±SE=0.579±.165, *t*(24)=3.849, *p*=.002, whereas the simple slope of AUDIT Consumption did not differ significantly from null in all other cells of the alcohol sensitivity group x sex interaction, *b*±SE=−0.175-(−0.061)±.125–0.165, *t*(24)=0.485–1.326, *p*=.197–0.632.

#### 3.2.4. Exploratory ERP-BOLD association analyses

A significant MLR model containing the EEG-ERP measure of alcohol cue incentive salience was found for ACR in l-vlPFC BOLD: *R*^2^=0.242, *F*(3, 28)=2.984, *p*=.048. This MLR model contained a significant sex x EEG-ERP interaction (*p*=.047). However, this MLR model did not contain the alcohol sensitivity group x sex interaction effect detected in l-vlPFC when testing the alcohol sensitivity hypothesis, so the sex x EEG-ERP interaction effect detected in these exploratory analyses may not be robust. Consequently, it is presented only in Supplemental Information. Briefly: the previously collected EEG-ERP measure of alcohol cue incentive salience was positively associated with ACR in l-vlPFC BOLD among males but not females.

## 4. Discussion

This fMRI pilot study tested the idea that amplified affective-motivational reactivity to alcohol cues among people with LS to alcohol reflects heightened ACR in the mesocorticolimbic system. Alcohol sensitivity group differences were detected on ACR in l-cmOFC, l-vlPFC, l-putamen, and l-SN; however, many of these group differences depended on between-person differences in alcohol use, and one depended also on biological sex. Below, we discuss all observed alcohol sensitivity effects and the need for larger-scale neuroimaging investigations. We then consider the implications of a potential frontocortical-subcortical network basis for heightened ACR as a mechanism of LS-based AUD risk. Finally, we discuss limitations and offer concluding remarks.

### 4.1. Alcohol sensitivity group differences on neural ACR

In l-vlPFC, ACR was greater for group LS than HS, and this difference was not moderated by any other variables (e.g., alcohol use or biological sex). Based on the proposed role of the vlPFC in attentional, behavioral, and mnemonic control [73-75], l-vlPFC hyperreactivity to alcohol cues among people with LS suggests that alcohol cues may be eliciting more overt (presumably positive) evaluative responses from, or promoting more higher-order elaborative processing (e.g., retrieval of long-term alcohol-associated memories into working memory, manipulation of semantic information in working memory), in people with LS than HS to alcohol. Furthermore, l-vlPFC hyperreactivity to alcohol cues among people with LS to alcohol suggests a prefrontocortical contribution to the amplified affective-motivational reactivity to alcohol cues associated with the LS phenotype. This possibility is consistent with the proposed role of prefrontocortical structures in aberrant drug cue reactivity [76] and incentive salience [77].

In l-cmOFC, ACR was jointly determined by alcohol sensitivity group and AUDIT Consumption or Problem subscale scores, and effects were stronger for Problem than Consumption scores. At lower AUDIT scores, indicating lighter or less problematic alcohol use, ACR in l-cmOFC was greater in group HS compared to group LS. However, as AUDIT scores increased, indicating heavier or more problematic alcohol use, ACR in l-cmOFC increased in group LS and decreased in group HS. The divergent relationship of ACR in l-cmOFC to alcohol use or problem level may reflect the proposed role of the mOFC in the computation or representation of cue-based expectations about the hedonic impact of the depicted reward [78,79]. mOFC hyperreactivity to alcohol cues among people with LS to alcohol who also endorse heavier or more problematic alcohol use is consistent with the proposal that LS to alcohol confers risk for AUD via incentive sensitization to alcohol and its associated cues across the alcohol use career [15]. Among people with LS, alcohol cues may become increasingly or preferentially able to activate positive alcohol use-outcome expectancies (i.e., positive expected hedonic impact) across the alcohol use career due to incentive sensitization. In contrast, among people with HS to alcohol, alcohol cues may become increasingly or preferentially able to active negative alcohol use-outcome expectancies (i.e., negative expected hedonic impact) across the alcohol use career, which could account for blunted mOFC reactivity to alcohol cues among people with HS to alcohol who endorse heavier or more problematic alcohol use. Nonetheless, the ability of alcohol cues to differentially activate positive and negative alcohol use-outcome expectancies as a function of alcohol sensitivity phenotype remains to be tested.

In l-putamen, ACR was jointly determined by alcohol sensitivity group and AUDIT Consumption scores. At lower AUDIT Consumption scores, indicating lighter alcohol use, l-putamen ACR scores in group LS tended to be negative, indicating enhanced reactivity to control cues relative to alcohol cues, or null (i.e., near zero), indicating no differentiation between alcohol and control cues. However, as AUDIT Consumption scores increased, indicating heavier alcohol use, l-putamen ACR scores became increasingly positive and greater than null in group LS. In group HS, no link was detected between AUDIT Consumption scores and l-putamen ACR scores, which remained null or negative. As such, when alcohol use levels are similarly low, alcohol cues appear to elicit similar or less activation across the l-putamen for people in group LS than HS, but as alcohol use levels increase, ACR in l-putamen increases for those in group LS only. This alcohol sensitivity-related divergence in the relationship of ACR in l-putamen to alcohol use level parallels the divergence in the relationship of ACR in l-cmOFC to alcohol use or problem level. The human and non-human primate putamen or dorsolateral neostriatum in rodents is a nucleus of the basal ganglia that is important for the vigor of incentive responses to cues [80] as well as learning and expression of cue- or context-bound motor "habits" [81,82], including ritualized alcohol and drug seeking behavior [83-85]. Thus, the selective hyperreactivity of the putamen to alcohol cues with increasing alcohol use level in group LS is consistent with the proposal that LS to alcohol confers risk for AUD via incentive sensitization to alcohol and its associated cues across the alcohol use career [15]. In contrast, the lack of a detected link between putamen reactivity to alcohol cues and alcohol use levels in group HS is consistent with the protective nature of the HS phenotype.

In l-SN, ACR to alcohol cues was a function of alcohol sensitivity group, sex, and AUDIT Consumption scores. At lower AUDIT Consumption scores, indicating lighter alcohol use, l-SN ACR scores tended to be negative, indicating enhanced reactivity to control cues relative to alcohol cues, for group LS males. However, as AUDIT Consumption scores increased, indicating heavier alcohol use, l-SN ACR scores became increasingly positive and greater than null for group LS males. In contrast, no link was detected between AUDIT Consumption scores and l-SN ACR in group LS females, group HS females, or group HS males. The l-SN ACR scores for most people in these groups were null, indicating no differentiation between alcohol and control cues; however, there were cases in each group with positive as well as negative l-SN ACR scores. The proposal that LS to alcohol confers risk for AUD via incentive sensitization does not make predictions about the role of biological sex [15]. However, heightened ACR in l-SN among heavier drinking males with LS to alcohol suggests that biological sex may be an important moderator of LS-based risk for AUD. The current study was not powered to detect potential differences in neural ACR due to sex as a biological variable, and gender identity was not assessed. Yet, alcohol sensitivity and alcohol use can differ by sex due to differences in pharmacokinetics (e.g., females tend to have less total body water leading to higher blood alcohol concentrations per drink) [86]. Additionally, alcohol use can differ as a function of gender identity due to sociocultural norms (e.g., in Western cultures, it is more socially acceptable for men than women to use alcohol, and excessive alcohol use remains associated with traditional conceptualizations of masculinity) [87,88]. Given that gender identity and biological sex may strongly shape alcohol use and its consequences, including neurofunctional changes, across the lifespan, these factors should be considered in future large-scale fMRI studies of ACR.

Finally, it is important to emphasize joint effects of alcohol sensitivity and alcohol use or problem level on neural ACR underscore the need for future large-scale neuroimaging studies to disentangle their unique effects. It is not surprising that alcohol use or problem level moderated all but one of the observed alcohol sensitivity group effects on neural ACR in the current study because the alcohol sensitivity groups differed in terms of alcohol use and problem level (as expected [7,9]). Statistical estimates of the unique effects of alcohol sensitivity and alcohol use or problem level in future large-scale studies may benefit from uniform sampling across the distributions of alcohol sensitivity and alcohol use or problem level. Strategic over-sampling of "rare" cases (e.g., people with LS to alcohol who endorse light alcohol use, people with HS to alcohol who endorse heavier or more problematic alcohol use) also may be useful. Furthermore, longitudinal studies spanning the developmental trajectory of alcohol use will be critical for isolating the effects of sensitivity vs. use on neural ACR because sensitivity and use may covary over time (e.g., innate LS to alcohol may promote heavier use, but heavier use itself may lead to acquired LS to alcohol [i.e., tolerance]) [89]. Longitudinal studies of neural ACR also are important because incentive sensitization and other theorized neurobiological mechanisms of the addiction cycle posit progressive accumulation functional neuroadaptations with chronic use [90,91], which should be evident as within-person changes in neural ACR and may be obscured in case-control studies by high between-person variability in neural ACR.

### 4.2. Implications of heightened ACR network for LS-based AUD risk

Trait-like measures of alcohol craving, consumption, and problems were elevated in group LS, as expected based on prior work that has established the LS phenotype as a risk factor for AUD [1,10]. Heightened neural ACR in the l-cmOFC, l-vlPFC, and l-putamen among people with the LS to alcohol who also endorsed heavy or problematic alcohol use suggests alcohol cue hyperreactivity in one of two parallel cortico-striatal loops through the basal ganglia (or both). The first cortico-striatal loop, represented here by l-cmOFC and l-putamen, is the so-called "affective" or "limbic" cortico-striatal loop for emotional and motivational responses to cues/contexts [43,92,93]. The second cortico-striatal loop, represented here by l-vlPFC and l-putamen, is the so-called "associative" or "cognitive" cortico-striatal loop for goal-based, rapid strategic selection among competing cue/context-related action plans [43,92,93]. Both of these cortico-striatal loops are modulated by the ascending mesocorticolimbic dopamine system [93]. Potential hyperreactivity of these cortico-striatal loops to alcohol cues among people with LS to alcohol who also endorsed heavy or problematic alcohol use suggests that neural activity in frontocortical and subcortical structures alike contributes to the oft-observed amplified affective-motivational reactivity to alcohol cues among people with LS to alcohol and, thereby, LS-based risk for AUD. Future large-sample fMRI studies could use the l-cmOFC and l-vlPFC as seeds for functional connectivity analyses and examine the extent to which the "affective" and "cognitive" cortico-striatal loops are selectively engaged in the alcohol cue reactivity task-state (relative to resting-state) for LS compared to HS individuals. If affective/cognitive cortico-striatal loop hyper-reactivity to alcohol cues is assessed early in the alcohol use career of individuals with LS or HS to alcohol, its ability to forecast the onset or progression of AUD symptomatology could be tested *via* longitudinal follow-up on alcohol use. An additional strategy would be to employ a data-driven approach (e.g., functional connectivity multivariate pattern analysis [fc-MVPA] [94]) to elucidate the neural circuitry mediating alcohol sensitivity phenotype-based differences in alcohol cue reactivity and test the predictive utility of alcohol cue-induced activity in that elucidated neural circuit, which may or may not include the "affective" and "cognitive" cortico-striatal loops.

### 4.3. Limitations

The study’s findings are tempered by several limitations. The primary limitation is the small overall sample size and limited number of persons per alcohol sensitivity group. However, while they should be interpreted with caution, these novel preliminary findings are compelling and may inform future large-scale investigations into the neurobiological loci of differential alcohol cue reactivity as a function of alcohol sensitivity phenotype. A second limitation is that the analytic approaches used here can reveal only between-group differences in the *level* of activation in brain areas found to be differentially responsive to alcohol cues at the sample level (i.e., in both groups). Thus, whether alcohol cues might activate different areas in individuals reporting HS vs. LS to alcohol remains an open question that requires a larger sample to assess. A third limitation is that reactivity to alcohol/drug images was defined in relation to reactivity to affectively neutral complex images (i. e., ACR=Alc-CC). Although such contrasts are widely used in the alcohol/drug cue fMRI literature [44,50,51,72,95], it is important to note that alternative contrasts (e.g., alcohol/drug images vs. non-alcohol/drug reward images) may be better suited for delineating neural mechanisms or biomarkers of addiction [96,97]. Here, these alternative contrasts (e.g., Alc-FD) did not return voxel clusters that survived family-wise error (FWE) correction to *p*<.05. A fourth limitation is that the use of a multiple linear regression (MLR) model fitting approach to test the alcohol sensitivity hypothesis on person-level ACR contrast beta coefficients involves testing multiple nested models in order to arrive at the best-fitting model yet no correction for multiple testing was applied. Although multiple testing correction is not typically applied to a model search process, false positives from multiple testing remain a concern. A fifth limitation concerns the test of association between the previously collected EEG-ERP measure of alcohol cue incentive salience and fMRI BOLD ACR.

Specifically, there was considerable between-person variation in the amount of time elapsed between EEG and fMRI visits (Median: 0.80 yr, Range: 0.20–3.12 yr) since the fMRI study opportunity was not planned as part of the parent study. This variable interval would be expected to diminish statistical power to detect a BOLD-ERP association. Relatedly, the ERP measure was obtained from a cue reactivity task that differs considerably from the task used for fMRI, which also would be expected to diminish the ability to detect BOLD-ERP correlations.

A sixth limitation pertains to the ACR cluster in the l-cmOFC, the peak voxel of which was located in the gyrus rectus. Signal dropout is a serious concern in the gyrus rectus due to its proximity to sinuses. Although we verified that all participants in the study had complete data in this region for the Alc and CC conditions constituting the ACR contrast, signal dropout may have limited the extent of signal: 12 out of 32 participants (47 % of the sample) had an Alc or CC condition *t*-score between −0.5 and +0.5 at the peak voxel. Signal dropout may thus limit the reproducibility of findings involving the l-cmOFC. Finally, it is important to note that the post-hoc subcortical atlas-based analyses averaged the BOLD response across the entire volume of each structure. This means the current study cannot localize the alcohol sensitivity-related effects on ACR within subcortical structures (e.g., l-putamen, l-SN). However, subcortical structures are as intricately (albeit differently) organized as their cortical partners [92,98,99], so localizing ACR effects more precisely within specific subcortical structures would be a major contribution of future large-scale fMRI studies of ACR.

There also are important limitations related to sample characteristics. First, the sample was comprised of primarily non-Hispanic White emerging adult college students. Thus, findings can be expected to generalize to other college-educated Non-Hispanic White emerging adults in the U.S. but may not generalize to emerging adults from other ethnic/racial (e.g., Black, Hispanic/Latinx) or disadvantaged backgrounds. Second, inclusion criteria for recruitment used a liberal threshold for alcohol use (at least monthly alcohol use in the past year and at least 1 binge-drinking episode in the past 6 months). Third, exclusion criteria for recruitment included a history of unsuccessful attempts to quit or moderate alcohol use, but did not include other addictive substance use. Relatedly, neither AUD nor other substance use disorders (SUDs) were formally assessed as part of the current study (e.g., using a semi-structured clinical interview). Thus, the current study’s findings may reflect participants’ relatively short history and limited extent of alcohol use as well as unknown AUD/SUD diagnostic status. This may explain why ACR clusters were not detected in subcortical nodes of the mesocorticolimbic system in either the mesocorticolimbic system-masked or exploratory whole-brain level-2 model of the fMRI BOLD response. Previous fMRI studies in clinical samples suggest that much heavier and/or more disordered alcohol use may be required for subcortical nodes of the mesocorticolimbic system to become hyperre-active to alcohol cues [45,50,51,100-102].

### 4.4. Conclusions

In conclusion, the current study points to amplified ACR in cortical (cmOFC, vlPFC) and striatal (putamen) nodes of the mesocorticolimbic system among persons reporting LS to alcohol, especially those who also report heavy or problematic alcohol use. This is significant, as it has been proposed that LS confers risk for alcohol misuse and AUD *via* incentive sensitization, which is believed to take place in the mesocorticolimbic system. This pilot fMRI study is the first to test potential differences in mesocorticolimbic system ACR among persons reporting LS vs. HS to alcohol. However, fMRI studies with larger samples are needed to determine conclusively the neurobiological bases of amplified affective-motivational reactivity to alcohol cues as a function of LS to alcohol.

## Supplementary Material

1

## Figures and Tables

**Table 1:** Sociodemographic characteristics.

	HS	LS	
	*n*	*n*	LS=!=HS?
			X^2,^ df, *p*
Female	9	9	
Ethnicity			
Hispanic	0	0	
Race			2.13, 2, 0.344
Asian	1	0	
Black	0	0	
White	14	16	
Multiple Selected	1	0	
Handedness			0, 1, 1
Right-Handed	14	15	
Socioeconomic Status Indicators			
Completed Education			1.69, 2, 0.429
High school diploma or GED	3	1	
Some college or vocational training	11	14	
Bachelor’s degree	2	1	
Current Educational Status			3.03, 3, 0.386
Not enrolled in any schooling	0	1	
Enrolled in 2-year college	0	1	
Enrolled in 4-year college	15	14	
Enrolled in graduate school	1	0	
Current Housing Status			16.71, 5, 0.005
Residence hall on campus	4	1	1.8, 1, 0.180
Fraternity/sorority house on campus	0	4	4, 1, 0.045
With family, off-campus	0	2	2, 1, 0.157
With close friends, off-campus	1	6	3.57, 1, 0.059
With roommates, off-campus	10	2	5.33, 1, 0.021
Alone, off-campus	1	1	
Employment Status			1.58, 4, 0.812
Employed full-time	0	0	
Employed part-time (<30 hr/wk)	11	10	
Self-employed	1	2	
Not employed, seeking work	2	3	
Not employed, unable to work	1	0	
Prefer not to say	1	1	
Personal Pre-Tax Income (USD)			1.04, 2, 0.595
40k-60k	0	1	
20k-40k	0	0	
<20k	14	13	
Prefer not to say	2	2	
	M (SD)	M (SD)	LS=!=HS?
			*U, p*
Age, yr	20.31 (1.19)	20.37 (1.20)	126, 0.968
BMI, kg/m^2^	26.64 (6.59)	24.57 (3.34)	138, 0.720

*Note*. Total *N* = 32. USD=United States of America Dollar.

**Table 2:** Alcohol sensitivity, alcohol use, and alcohol craving by alcohol sensitivity group.

	HS	LS	LS=!=HS?
	M (SD)	M (SD)	*U, p*
*Alcohol sensitivity*			
ASQ Light	2.73 (1.10)	4.69 (3.23)	33, <0.001
ASQ Heavy	7.10 (2.77)	11.33 (3.23)	39, <0.001
ASQ Total	4.48 (1.73)	7.35 (1.82)	33, <0.001
*Alcohol use*			
Years Since First Alc. Intox.	2.35 (1.17)	3.38 (1.17)	64, 0.026
Years Since Reg. Alc. Use	2.36 (1.09)	2.25 (0.95)	140, 0.665
Drinking days per week (past year)	1.30 (1.10)	3.09 (1.61)	41, 0.001
Drinks per drinking day (past year)	3.56 (1.49)	6.78 (3.17)	48, 0.002
Max drinks in 24 hr (past year)	9.81 (6.10)	19.75 (4.71)	20, <0.001
Max drinks in 24 hr (lifetime)	13.94 (8.16)	25.06 (6.86)	33, <0.001
Drinking days (past week)	1.69 (1.54)	3.25 (2.38)	76, 0.049
Drinks per drinking day (past week)	2.18 (1.08)	5.72 (2.62)	16, <0.001
Problems per drinking day (past week)	0.04 (0.14)	0.21 (0.32)	55.5, 0.065
AUDIT Consumption	4.31 (1.74)	7.75 (1.81)	23.5, <0.001
AUDIT Problem	2.50 (3.01)	8.00 (5.73)	41.5, 0.001
AUDIT Total	6.81 (4.09)	15.75 (7.23)	29.5, <0.001
*Alcohol craving (past week)*			
CEQ-F Intensity (frequency)	6.94 (4.19)	12.44 (5.37)	41, 0.001
CEQ-F Imagery	9.12 (5.17)	13.19 (8.82)	95.5, 0.226
CEQ-F Intrusiveness	0.94 (2.72)	2.94 (6.17)	85, 0.051
CEQ-F Total	17 (9.88)	28.56 (17.10)	61, 0.012
CEQ-S Intensity (max intensity)	11.19 (5.52)	16.5 (6.20)	65, 0.018
CEQ-S Imagery	12.25 (9.15)	15.56 (8.85)	105.5, 0.405
CEQ-S Intrusiveness	3.81 (5.43)	5.62 (6.83)	107, 0.419
CEQ-S Total	27.25 (18.43)	37.69 (18.70)	81.5, 0.083
*Alcohol craving (momentary)*			
Pre-Cue Reactivity Task	3.69 (0.60)	3.61 (1.77)	150, 0.385
Post-Cue Reactivity Task	3.81 (1.33)	4.27 (2.29)	110, 0.501
Change in Craving (Post-Pre)	0.12 (1.46)	0.65 (2.77)	123, 0.876

*Note. N* = 16/group. ASQ=Alcohol Sensitivity Questionnaire. AUDIT=Alcohol Use Disorders Identification Test. CEQ-F=Craving Experiences Questionnaire Frequency form. CEQ-S=Craving Experiences Questionnaire Strength form.

**Table 3:** Reward cue-reactive regions identified by different cue type contrasts in 2nd level fMRI model focused on the mesocorticolimbic system.

Contrast	Cluster	Cluster Size (# voxels)	Activation Volume (mm^3^)	MNI Coordinates (X Y Z) for Peak Voxel	Anatomical Area of Peak Voxel
Alc > CC					
	1	34	115	−6 59 −6	left frontal middle orbital gyrus (left rostral medial orbitofrontal cortex [L-rmOFC])
	2	37	125	−3 34 −16	left rectus (left caudal inferior medial orbitofrontal cortex [L-cmOFC])
	3	19	64	−45 28 5	left frontal inferior gyrus - triangularis (left ventrolateral prefrontal cortex [L-vlPFC])
F/D > CC					
	–	–	–	–	

Note. Alc=alcohol cues. F/*D*=non-alcohol food and drink cues. CC=affectively neutral complex control cues. MNI = Montreal Neurological Institute. In the Anatomical Area of Peak Voxel column, we present the specific area label taken from the Automated Anatomical Labeling (AAL) atlas [97] followed by the more general label for the same area in parentheses. In the manuscript, the more general area label is used. Activation volume = cluster size (# voxels) x voxel size (1.5 mm^3^).

## Data Availability

Data will be made available on request.
