# Supplementary material for: Mesocorticolimbic system reactivity to alcohol use-related visual cues as a function of alcohol sensitivity phenotype: A pilot fMRI study
Source: Addict Neurosci. Author manuscript; Available in PMC 2024 Jun 27. (PMC11209874; doi:10.1016/j.addicn.2024.100156)
Supplement: 1 [file NIHMS2001156-supplement-1.docx]

**Supplemental Information**

This supplemental material accompanies the article, "Mesocorticolimbic system reactivity to alcohol use-related visual cues as a function of alcohol sensitivity phenotype: a pilot fMRI study," by RU Cofresí, S Upton, AA Brown, TM Piasecki, BD Bartholow, & B Froeliger.

**Stimuli**

**Figure S1** shows example stimuli for the alcohol (Alc), food/drink (F/D), nicotine (Nic), complex control (CC), and simple control (SC) cue conditions.

The following jpg files from the International Affective Picture Set (IAPS) were used for the CC cue condition: 2026, 2036, 2056, 2359, 2374, 2384, 2393, 2394, 2398, 2487, 2495, 2635, 2880, 4612, 6150, 7002, 7011, 7019, 7020, 7021, 7032, 7034, 7040, 7042, 7043, 7050, 7056, 7060, 7061, 7090, 7150, 7165, 7170, 7175, 7179, 7185, 7211, 7217,7224, 7234, 7235, 7509, 7512, 7513, 7950, 5726, 7026, 5635, 7100, 7180, 7240, 7490, 7491, 7495, 7510, 7560, 7590, 7595, 7632, 9468.

**Method**

**Nicotine Use Behavior Assessment**

**Questionnaires**

***Self-Reported Nicotine Use***

Lifetime nicotine use was assessed. Individuals endorsing any nicotine use in their lifetime were then asked questions about which nicotine products they had consumed as well as whether they currently consumed any nicotine products. Follow-up on current users involved the Fagerstrom Test for Nicotine Dependence (FTND; [1]) if currently using tobacco cigarettes and the Penn State Electronic Cigarette Dependence Index (PSECDI; [2]) if currently using electronic nicotine delivery systems (ENDS); however, no participants endorsed current tobacco cigarette use, so only PSECDI data were obtained. ICR for PSECDI scores was fair (α=.69). Finally, at the end of the lab visit, participants indicated on an 8-day TLFB calendar on which days they used nicotine. Experimenters then reviewed the TLFB calendar with participants. For every day in the calendar on which nicotine use was indicated, experimenters asked the participant questions about the number of use episodes. Any days on the TLFB calendar on which participants indicated both alcohol and nicotine use were probed to determine whether any alcohol and nicotine use episodes on those days took place at the same time.

***Self-Reported Craving for Nicotine***

Past week's nicotine cravings were assessed using the Craving Experience Questionnaire (CEQ; [3]), which captures the frequency and strength of cravings on separate forms. ICR for alcohol CEQ frequency and strength form scores was good-to-excellent (α=.82-.95). ICR for nicotine CEQ frequency and strength form scores was good-to-excellent (α=.87-.96). In the scanner, momentary alcohol and nicotine cravings were assessed using single-item measures ("How much do you want [to drink alcohol/to vape] right now?"; response options ranged from "not at all" [1] to "a lot" [8] in 1-unit increments).

**Confirmatory Urinary Cotinine Analysis**

Self-reported use or non-use of nicotine was verified using cotinine urinalysis. Here, a Healgen OneStep COT dipcard was used to test for cotinine metabolites in a freshly produced urine sample (≈10-20 mL). Per the manufacturer (Healgen Scientific LLC, Houston, TX, USA), the Healgen OneStep COT test can detect cotinine concentrations as low as 200 ng/mL. Based on the rate at which cotinine is eliminated from circulation by the kidneys, the manufacturer claims positive Healgen OneStep COT tests indicate nicotine use within the past 48-72 hr.

**Exploratory EEG-ERP and fMRI BOLD Analyses**

The mean amplitude of the P3/LPP component of the EEG-ERP response to alcohol (Alc) and non-alcohol (NonAlc) beverage pictures was derived from an EEG recording at a previous laboratory visit. The EEG recording was made during an active picture viewing task in which participants were asked to identify, as accurately and as rapidly as possible, between Alc and NonAlc beverage pictures. The amplitude of the P3/LPP component of the EEG-ERP response was scored as the average voltage from 300 to 700 ms post-cue onset across a cluster of 9 electrodes over the occipitoparietal scalp (PZ, P3, P4, P7, P8, PO7, PO8, O1, O2). The amplitude of the P3/LPP component of the EEG-ERP response to a cue indexes that cue's affective-motivational significance to the individual [4]. For technical details (e.g., recording parameters, preprocessing pipeline), please refer to our recent publications from these EEG data [5,6].

**Results**

**Nicotine Use Behavior**

A similarly small number of persons in group HS (*n*=3 out of 16 [19%]) and group LS (*n*=6 out of 16 [37%]) endorsed current nicotine use, specifically, they endorsed the use of electronic nicotine delivery systems (ENDS; e.g., electronic cigarettes, nicotine vaporizer pens; X^2^[1]=.458, *p=*.498). Additionally, 1 person in group HS reported having recently tried 1 non-combustible nicotine product, a Zyn pouch, at a social drinking episode in the week before their study visit. Nicotine use or non-use self-report was verified via cotinine urinalysis.

**Table S1** shows that among nicotine co-users (*n*=9 [28% of sample]), nicotine use frequency (days) and intensity (times per day) in the past week were significantly elevated for group LS compared to HS. Nicotine dependence severity (as indexed by PSECDI scores) did not differ significantly by group. Past week's nicotine craving frequency (as indexed by CEQ-F Intensity scores) and intensity (as indexed by CEQ-S Intensity scores) also did not differ significantly by group. Craving for nicotine in the scanner was statistically similar between groups pre- and post-task, and within-person change scores did not differ significantly between groups.

**Table S1** also shows that the frequency of alcohol and nicotine co-use days in the past week also was significantly elevated in group LS compared to HS; however, the number of alcohol and nicotine co-use *episodes* on those days did not differ significantly between groups. Thus, the former group difference only reflects the higher alcohol use frequency (days) in group LS compared to HS, which was reported in **Table 2** (main text).

Observed differences in nicotine consumption patterns between current ENDS users in group HS and LS are interesting because it is possible that people with LS to alcohol also may have LS to nicotine (viz., there may be innate or acquired cross-tolerance). This possibility is worth exploring in future behavioral or questionnaire-based studies using larger samples enriched for alcohol and nicotine co-users.

**Nicotine Cue Reactivity (NCR) in the Alcohol Cue Reactivity fMRI Task**

Masked Second-Level Functional ROI Results:

No clusters were identified for NCR.

Exploratory Whole-Brain Second-Level Functional ROI Results:

As shown in **Table S2**, four clusters were identified for NCR. These were located in the left and right hemisphere fusiform gyrus and temporal middle gyrus. Although few participants in groups HS and LS endorsed current nicotine use, we applied a MLR model fitting strategy (following the MLR model fitting strategy described in the main text) to explore potential interactions between alcohol sensitivity and current nicotine use status and sex on NCR level. No significant MLR models of between-person variation in NCR level at the identified ROIs were found that contained a significant main effect of alcohol sensitivity or a significant interaction between alcohol sensitivity and nicotine use status or sex. The only significant MLR model found was one for NCR level at the superolateral peak voxel (MNI coordinates: x=-55, y=-68, z=11) in the NCR cluster located in the left hemisphere middle temporal gyrus (a.k.a., the left visual association area): *R*^2^=.140, *F*(1,30)=4.900, *p*=.035. This MLR model contained only a significant main effect of nicotine use status (*p*=.035) such that NCR level here was elevated for the 9 individuals in the sample endorsing current nicotine use (M±SE=1.353±0.229) compared to their 23 peers who did not endorse current nicotine use (M±SE=0.755±0.143): M_D_±SE_D_=0.599±0.270, *t*(30)=2.214, *p*=.035.

**Brain-Behavior Association Analyses Related to Alcohol Cue Reactivity (ACR) in the Alcohol Cue Reactivity fMRI Task: Detected Effects Not Relevant to the Alcohol Sensitivity Hypothesis**

Masked Second-Level Analysis Functional ROIs:

The best MLR models of between-person variation in ACR in L-cmOFC (*R*^2^=.347-.376, *F*[5, 26]=3.133-2.767, *p*=.024-.039) indicated the following significant 2-way interaction effects: (i) sex group x AUDIT Consumption (*p*=.019), and (ii) sex group x AUDIT Problem (*p*=.015). Decomposition indicated that the sex group x AUDIT Consumption and sex group x AUDIT Problem interaction effects were similar, which may reflect the large correlation between AUDIT Consumption and Problem scores (*r*=.76, *p*<.001), but also that the sex group x AUDIT Problem interaction effects were stronger. As shown in **Figure S3A-B**, the simple slope of AUDIT Consumption differed significantly between females and males (∆b±SE=.101±.040, *t*(26)=2.501, *p*=.019), as did the simple slope of AUDIT Problem (∆b±SE=.051±.020, *t*(26)=2.608, *p*=.015). In males, there was a significant, *negative* simple slope of AUDIT Problem, b±SE=-.042±.016, *t*(26)=2.639, *p*=.014, whereas in females, it did not differ significantly from null, b±SE=.010±.017, *t*(26)=0.551, *p*=.586. The corresponding simple slopes of AUDIT Consumption trended in the same directions, but were not significantly different from null in either sex group (*p*=.150-.175). This indicates that, among males, those who experienced more alcohol use-related problems showed greater ACR in the L-cmOFC BOLD response, whereas there was no such association among females.

Post-Hoc Subcortical Atlas-Based ROIs:

The best MLR models of between-person variation in ACR across the L-SN (*R*^2^=.351-.426, *F*[5-7, 24-26]=2.548-2.819, *p*=.036-.041) indicated a significant sex group x CEQ-S Intensity interaction effect (*p*=.013). The best MLR model of between-person variation in ACR across the R-SN (*R*^2^=.179, *F*[1, 30]=6.54, *p*=.016) contained only a significant main effect of CEQ-F Intensity (*p*=.016).

As shown in **Figure S4A**, decomposition of the sex group x Alcohol CEQ-Strength Intensity interaction effect on ACR in the L-SN BOLD response found that the simple slope of Alcohol CEQ-Strength Intensity differed significantly between females and males (∆b±SE=.115±.043, *t*(26)=2.658, *p*=.013). In males, there was a significant, *positive* simple slope of Alcohol CEQ-Strength Intensity, b±SE=.117±.035, *t*(26)=3.309, *p*=.003, whereas, in females, it did not differ significantly from null, b±SE=.002±.025, *t*(26)=0.086, *p*=.932. This indicates that, among males, those who experienced more intense alcohol craving experiences in the past week showed greater ACR in the L-SN BOLD response, whereas there was no such association among females.

As shown in **Figure S4B**, people reporting more frequent alcohol craving experiences in the past week (i.e., greater Alcohol CEQ-Frequency Intensity scores) showed greater ACR in the R-SN BOLD response. The simple slope was significant: b±SE =.067±.026, *t*(30)=2.557, *p*=.016.

**Confirmatory Analysis of Alcohol Cue Reactivity (ACR) in the ERP P3/LPP Response**

**Figure S5A** shows the ERP response to Alc and NonAlc cues for groups LS and HS. As shown in **Figure S5B**, in group LS, the P3/LPP amplitude was visibly greater for Alc compared to NonAlc cues, and the within-person comparison was significant, *t*(15)=2.15, *p*=.048. In contrast, there is no visible difference in P3/LPP amplitude for Alc and NonAlc cues in group HS, *t*(15)=0.70, *p*=.493. Additionally, as shown in **Figure S5B**, within-person ACR (i.e., the Alc - NonAlc P3/LPP amplitude difference score) was visibly greater in group LS (M±SE=1.51±0.70 µV) compared to HS (M±SE=0.471±0.67 µV), but the between-group comparison was not significant, *t*(30)=1.07, *p*=.146, unless we statistically controlled for between-person differences in P3/LPP amplitude due to sex/gender and typical alcohol use frequency across the year preceding the lab visit: *t*(28)=1.71, *p*=.049. Nonetheless, the overall pattern of results here is consistent with our previous demonstrations, in much larger samples, that the *lower* the self-reported sensitivity to acute alcohol, the *larger* the ACR in the P3/LPP [5,7–9].

**Association between ACR in ERP P3/LPP response and ACR in fMRI BOLD response**

Mesocorticolimbic masked second-level analysis ROIs:

As shown in **Figure S6**, decomposition of the sex group x ACR in ERP P3/LPP interaction effect on ACR in the L-vlPFC BOLD response found that the simple slope of ERP P3/LPP differed significantly between females and males (∆b±SE=.086±.042, *t*(28)=2.073, *p*=.047). In males, there was a significant, *positive* simple slope of ERP P3/LPP, b±SE=.093±.034, *t*(28)=2.749, *p*=.010, whereas, in females, it did not differ significantly from null, b±SE=.007±.024, *t*(28)=0.271, *p*=.788. This indicates that, among males, those who exhibited greater ACR in the ERP P3/LPP response at the previous visit showed greater ACR in the L-vlPFC BOLD response in the current study, whereas there was no such association among females.

Whole-brain exploratory second-level analysis ROIs:

No MLR models containing the ERP P3/LPP measure were significant.

Post-hoc anatomically defined subcortical ROIs:

No MLR models containing the ERP P3/LPP measure were significant.

Discussion:

The primary reason to expect an EEG-fMRI correlation here is that both measures are putatively indexing incentive salience attribution to alcohol cues or ACR more broadly. That is, to the extent both measures index the same construct, they theoretically may be associated. Similar EEG-fMRI correlations have been found for measures of reward anticipation (Pfabigan et al., 2014) and reward receipt (Carlson et al., 2011). The second reason is that Hanlon et al. (2014) found in their meta-analysis that, across ACR and other drug cue reactivity fMRI studies, there is enhanced BOLD reactivity to alcohol and other drug cues in parietal and occipital cortices associated with the visual sensory-perceptual system. ACR in the form of enhanced ERP P3 response amplitude at the previous lab visit may thus reflect enhanced ACR in vision-related posterior cortices. These two reasons provide a basis for predicting an association between EEG-fMRI; however, we acknowledge that it is unclear why ACR in the ERP P3 response amplitude at the previous lab visit would be directly associated with ACR in the L-vlPFC BOLD response but not with ACR in the vision-related posterior cortex BOLD activations that were observed in the current study. One possibility is that maximal between-person variation in the level of ACR occurs in different neural structures depending on the measurement task or paradigm, which we note in the Limitations section of the Discussion differed for EEG vs fMRI data collection (see main text).

**References**

1. Heatherton TF, Kozlowski LT, Frecker RC, Fagerstrom K. The Fagerstrom Test for Nicotine Dependence: a revision of the Fagerstrom Tolerance Questionnaire. Addiction. 1991;86:1119–1127.

2. Foulds J, Veldheer S, Yingst J, Hrabovsky S, Wilson SJ, Nichols TT, et al. Development of a questionnaire for assessing dependence on electronic cigarettes among a large sample of ex-smoking e-cigarette users. Nicotine Tob Res. 2015;17:186–192.

3. May J, Andrade J, Kavanagh DJ, Feeney GFX, Gullo MJ, Statham DJ, et al. The Craving Experience Questionnaire: A brief, theory-based measure of consummatory desire and craving. Addiction. 2014;109:728–735.

4. Hajcak G, Foti D. Significance?... Significance! Empirical, methodological, and theoretical connections between the late positive potential and P300 as neural responses to stimulus significance: An integrative review. Psychophysiology. 2020;57:1–15.

5. Cofresí RU, Piasecki TM, Bartholow BD. Acute sensitization of the P3 event-related potential response to beverage images and the risk for alcohol use disorder. Addict Neurosci. 2022;4:100041.

6. Cofresí RU, Piasecki TM, Hajcak G, Bartholow BD. Internal consistency and test–retest reliability of the P3 event‐related potential (ERP) elicited by alcoholic and non‐alcoholic beverage pictures. Psychophysiology. 2022;59.

7. Bartholow BD, Henry EA, Lust SA. Effects of Alcohol Sensitivity on P3 Event-Related Potential Reactivity to Alcohol Cues. Psychol Addict Behav. 2007;21:555–563.

8. Bartholow BD, Lust SA, Tragesser SL. Specificity of P3 Event-related potential reactivity to alcohol cues in individuals low in alcohol sensitivity. Psychol Addict Behav. 2010;24:220–228.

9. Martins JS, Bartholow BD, Lynne Cooper M, Irvin KM, Piasecki TM. Interactive Effects of Naturalistic Drinking Context and Alcohol Sensitivity on Neural Alcohol Cue-Reactivity Responses. Alcohol Clin Exp Res. 2019;43:1777–1789.

10. Pfabigan DM, Seidel EM, Sladky R, Hahn A, Paul K, Grahl A, et al. P300 amplitude variation is related to ventral striatum BOLD response during gain and loss anticipation: An EEG and fMRI experiment. Neuroimage. 2014;96:12–21.

11. Carlson JM, Foti D, Mujica-Parodi LR, Harmon-Jones E, Hajcak G. Ventral striatal and medial prefrontal BOLD activation is correlated with reward-related electrocortical activity: A combined ERP and fMRI study. Neuroimage. 2011;57:1608–1616.

12. Hanlon CA, Dowdle LT, Naselaris T, Canterberry M, Cortese BM. Visual cortex activation to drug cues: A meta-analysis of functional neuroimaging papers in addiction and substance abuse literature. Drug Alcohol Depend. 2014;143:206–212.

13. Tzourio-Mazoyer N, Landeau B, Papathanassiou D, Crivello F, Etard O, Delcroix N, et al. Automated anatomical labeling of activations in SPM using a macroscopic anatomical parcellation of the MNI MRI single-subject brain. Neuroimage. 2002;15:273–289.

| **Table S1** *Nicotine use and craving by alcohol sensitivity group* | | | |  |
| --- | --- | --- | --- | --- |
|  | HS (*n=*3) | LS (*n*=6) | LS=!=HS? | |
|  | M (SD) | M (SD) | *U*, *p* | |
| *Nicotine Use* |  |  |  | |
| Years Since Reg. ENDS Use | 3.67 (2.52) | 3.17 (1.33) | 11, .691 | |
| Use Days (past week) | 2.60 (3.05) | 6.44 (2.35) | 6, .022 | |
| Times Used Per Use Day (past week) | 2.47 (2.77) | 5.97 (3.61) | 7, .044 | |
| Use Days Also Used Alcohol (past week) | 1 (0) | 4.11 (2.47) | 2, .012 | |
| Alcohol Co-Use Episodes Per Day Used Both Alcohol and Nicotine (past week) | 1.50 (0.71) | 1.77 (2.81) | 10.5, .374 | |
| PSECDI | 6.00 (5.57) | 10.67 (3.14) | 4.5, .298 | |
| *Nicotine craving (past week)* |  |  |  | |
| CEQ-F Intensity (frequency) | 8.60 (8.59) | 12.78 (7.56) | 14, .283 | |
| CEQ-F Imagery | 7.40 (8.82) | 12.55 (13.71) | 16.5, .462 | |
| CEQ-F Intrusiveness | 3.40 (5.98) | 9.44 (5.88) | 9.5, .092 | |
| CEQ-F Total | 19.40 (22.07) | 34.78 (24.69) | 11.5, .161 | |
| CEQ-S Intensity (max intensity) | 12.80 (9.63) | 18.11 (7.54) | 13.5, .254 | |
| CEQ-S Imagery | 15.00 (13.26) | 17.78 (11.55) | 19, .688 | |
| CEQ-S Intrusiveness | 6.60 (10.94) | 11.89 (7.78) | 13.5, .256 | |
| CEQ-S Total | 34.49 (29.70) | 46.78 (24.13) | 13.5, .257 | |
| *Nicotine craving (momentary)* |  |  |  | |
| Pre-Cue Reactivity Task | 4.67 (1.15) | 3.85 (1.45) | 165, .104 | |
| Post-Cue Reactivity Task | 4.07 (1.18) | 4.30 (1.90) | 86.5, .105 | |
| Change in Craving (Post-Pre) | 0.00 (1.32) | 0.45 (2.16) | 84, .086 | |
| *Note*. ENDS=Electronic nicotine delivery systems (e.g., electronic cigarettes, nicotine vaporizer pens). Neither information about the specific ENDS used by participants (e.g., brand or device name) nor information about the concentration of nicotine in the liquid stock for the ENDS was collected. PSECDI=Penn State Electronic Cigarette Dependence Index. CEQ-F=Craving Experiences Questionnaire Frequency form. CEQ-S=Craving Experiences Questionnaire Strength form. | | | |  |

| **Table S2**  Reward cue-reactive regions identified by different cue type contrasts in exploratory whole brain 2nd level fMRI model | | | | | |
| --- | --- | --- | --- | --- | --- |
| Contrast | Cluster | Cluster Size (# voxels) | Activation Volume (mm^3^) | MNI Coordinates (X Y Z) for Peak Voxel | Anatomical Area for Peak Voxel |
| Alc > CC |  |  |  |  |  |
|  | 1 | 789 | 2663 | -44 -48 -22 | left fusiform gyrus |
|  |  |  |  | -40 -58 -19 | left fusiform gyrus |
|  |  |  |  | -40 -78 -10 | left occipital inferior gyrus |
|  | 2 | 54 | 182 | -46 -80 8 | left occipital middle gyrus |
|  | 3 | 52 | 176 | 40 -79 -13 | right occipital inferior gyrus |
|  | 4 | 39 | 132 | 42 -44 -19 | right fusiform gyrus |
|  | 5 | 9 | 30 | -6 50 -6 | left frontal middle orbital gyrus |
|  | 6 | 6 | 20 | -3 34 -16 | left rectus |
|  | 7 | 6 | 20 | -3 -50 24 | left posterior cingulum |
|  | 8 | 3 | 10 | -2 -32 34 | left posterior cingulum |
| F/D > CC |  |  |  |  |  |
|  | - | - |  | - | - |
| Nic > CC |  |  |  |  |  |
|  | 1 | 748 | 2525 | -48 -78 10 | left occipital middle gyrus |
|  |  |  |  | -44 -68 11 | left temporal middle gyrus |
|  |  |  |  | -56 -68 12 | left temporal middle gyrus |
|  | 2 | 284 | 959 | -44 -46 -18 | left fusiform gyrus |
|  |  |  |  | -42 -56 -18 | left fusiform gyrus |
|  | 3 | 394 | 1330 | 51 -67 8 | right temporal middle gyrus |
|  |  |  |  | 42 -60 -8 | right temporal middle gyrus |
|  | 4 | 70 | 236 | 40 -55 -14 | right fusiform gyrus |
|  |  |  |  | 42 -48 -19 | right fusiform gyrus |
| Note. Alc=alcohol cues. F/D=non-alcohol food and drink cues. Nic=nicotine cues. CC=complex affectively neutral control cues. MNI=Montreal Neurological Institute. Anatomical areas are taken from the Automated Anatomical Labeling (AAL) atlas [13]. Activation volume = cluster size (# voxels) x voxel size (1.5 mm^3^). | | | | | |

**Figure S1. Example Stimuli from Cue Reactivity Task**

| Alc | F/D | Nic (ENDS) | CC | SC |
| --- | --- | --- | --- | --- |
| 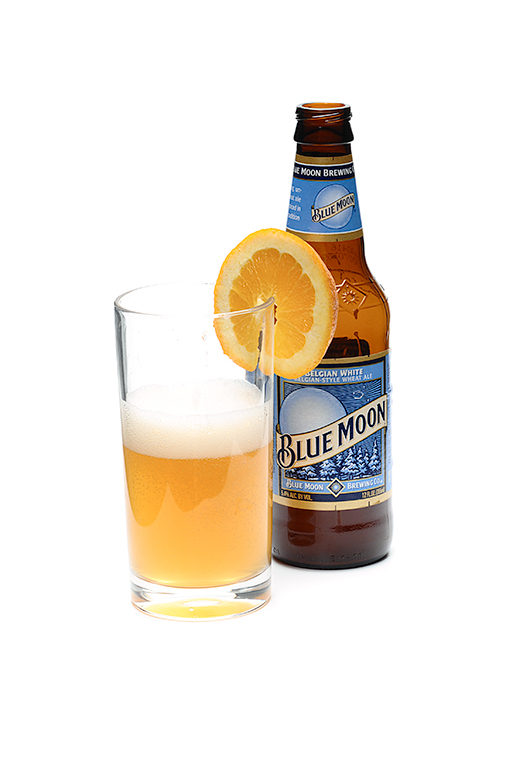 | 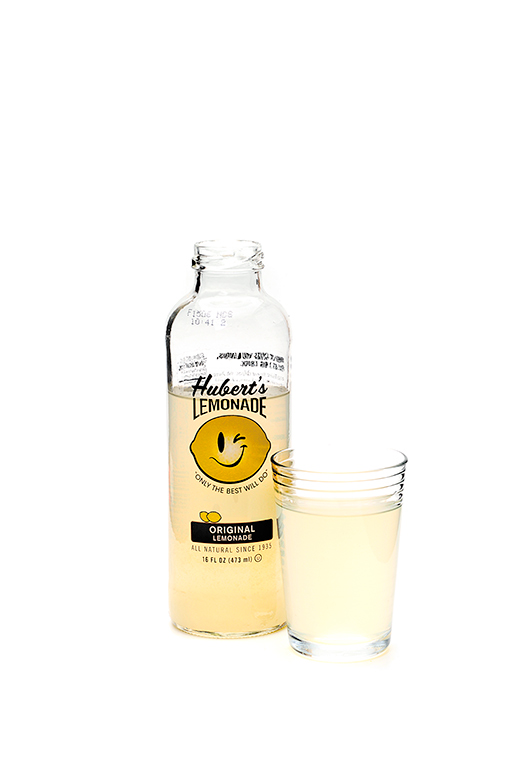 | 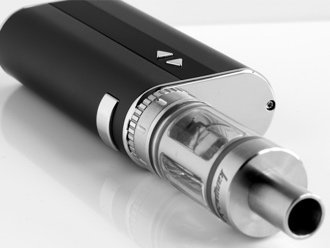 | 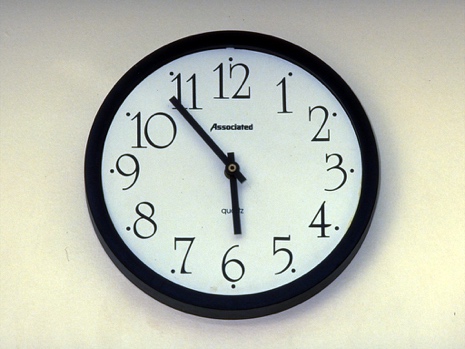 | 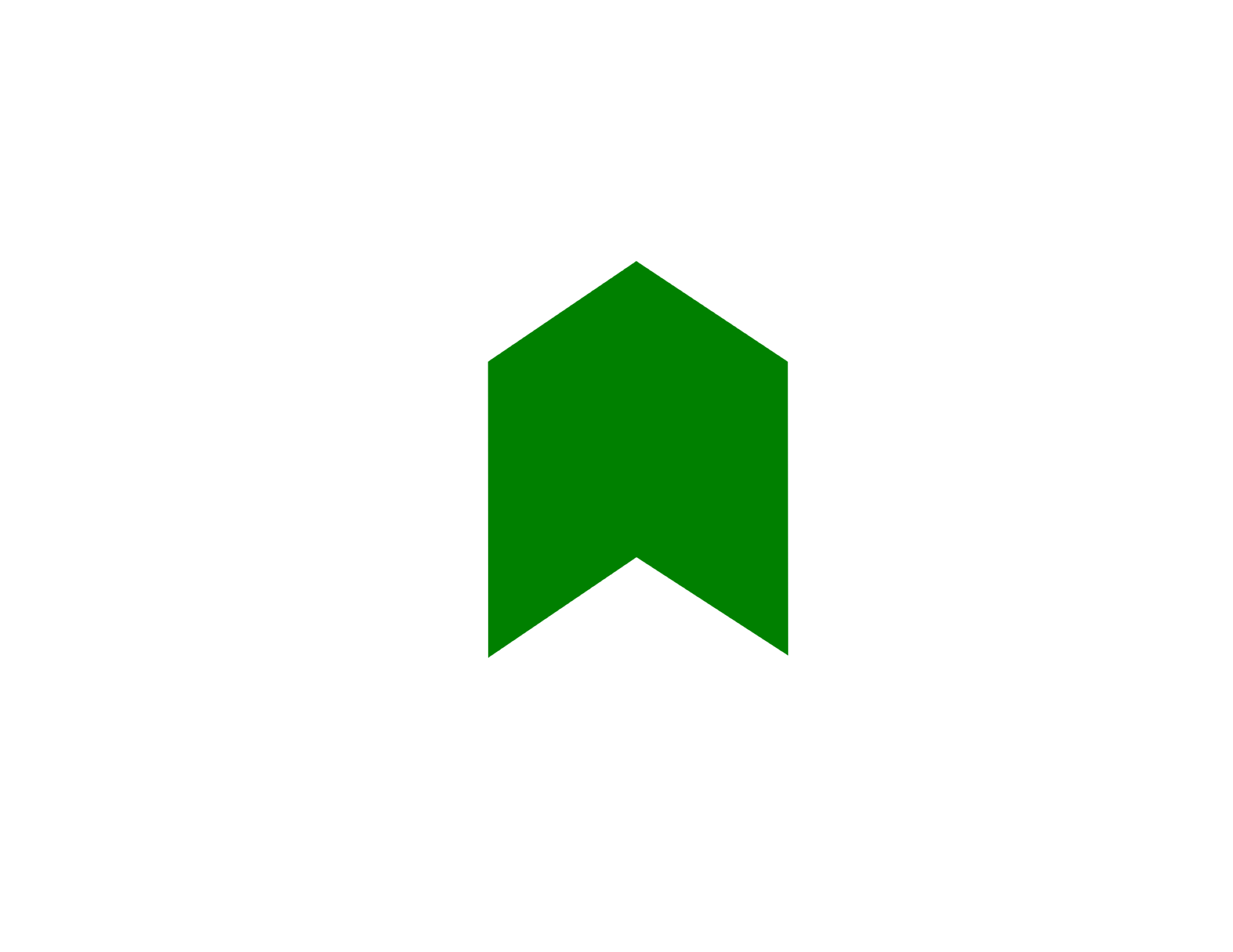 |
| 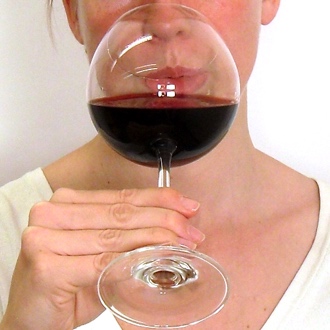 | 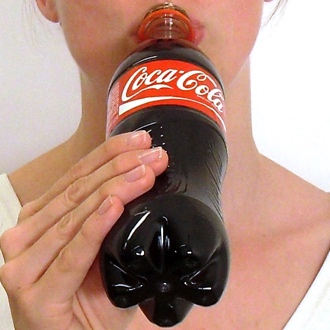 | 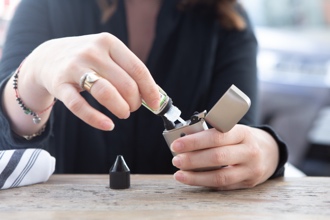 | 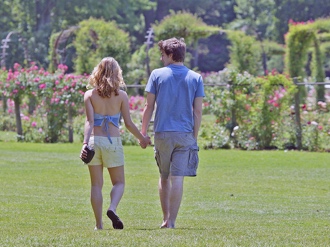 | 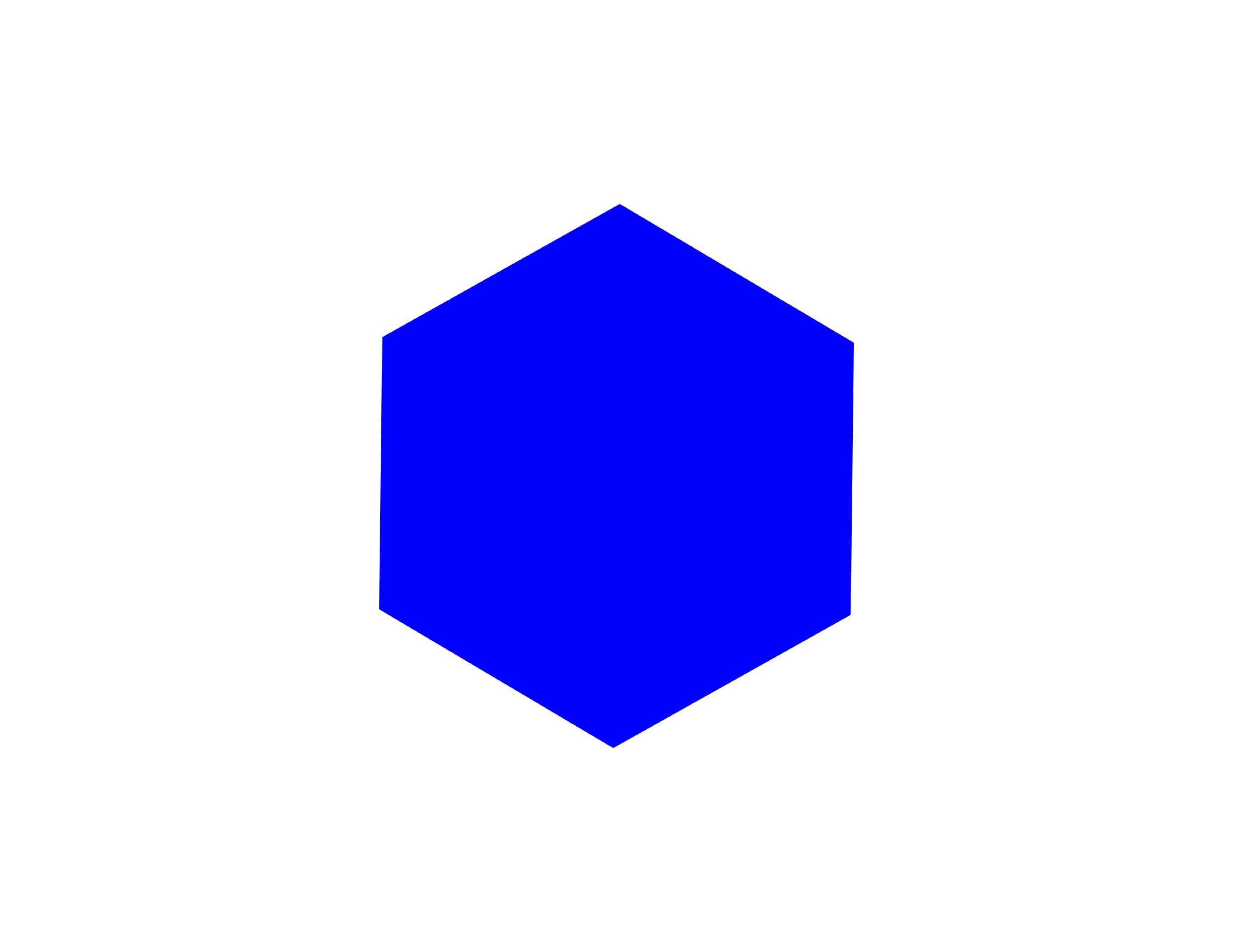 |

*Note*. Pictures in the alcohol (Alc), food/drink (F/D), nicotine (Nic), complex control (CC), and simple control (SC) cue conditions are shown. ENDS=electronic nicotine delivery systems. The Nic (ENDS) cue stimulus set included: 2 depictions of a 1^st^ generation ENDS (i.e., disposable e-cigarettes that strongly resemble tobacco cigarettes in their look and feel; a.k.a., "cigalikes"), 15 depictions of 2^nd^ generation ENDS (i.e., pen-style e-cigarettes with pre-filled or refillable cartridges with limited resemblance to tobacco cigarettes), 36 depictions of 3^rd^ generation ENDS (i.e., "tanks" or "mods"; modifiable product with large refillable tanks that do not resemble tobacco cigarettes; e.g., Kanger or INNOKIN vape kits; see example Nic cue [top]), and 7 depictions of 4^th^ generation ENDS (i.e., "pod mods"; modifiable product with pre-filled or refillable pod" cartridge that does not resemble to tobacco cigarettes; e.g., the JUUL pod system; see example Nic cue [bottom]). For more details about different generation ENDS, see: https://www.cdc.gov/tobacco/basic_information/e-cigarettes/pdfs/ecigarette-or-vaping-products-visual-dictionary-508.pdf

**Figure S2.** Brain areas that exhibited alcohol cue-specific reactivity (ACR) in the exploratory whole-brain but not mesocorticolimbic system-masked 2nd-level fMRI BOLD analysis


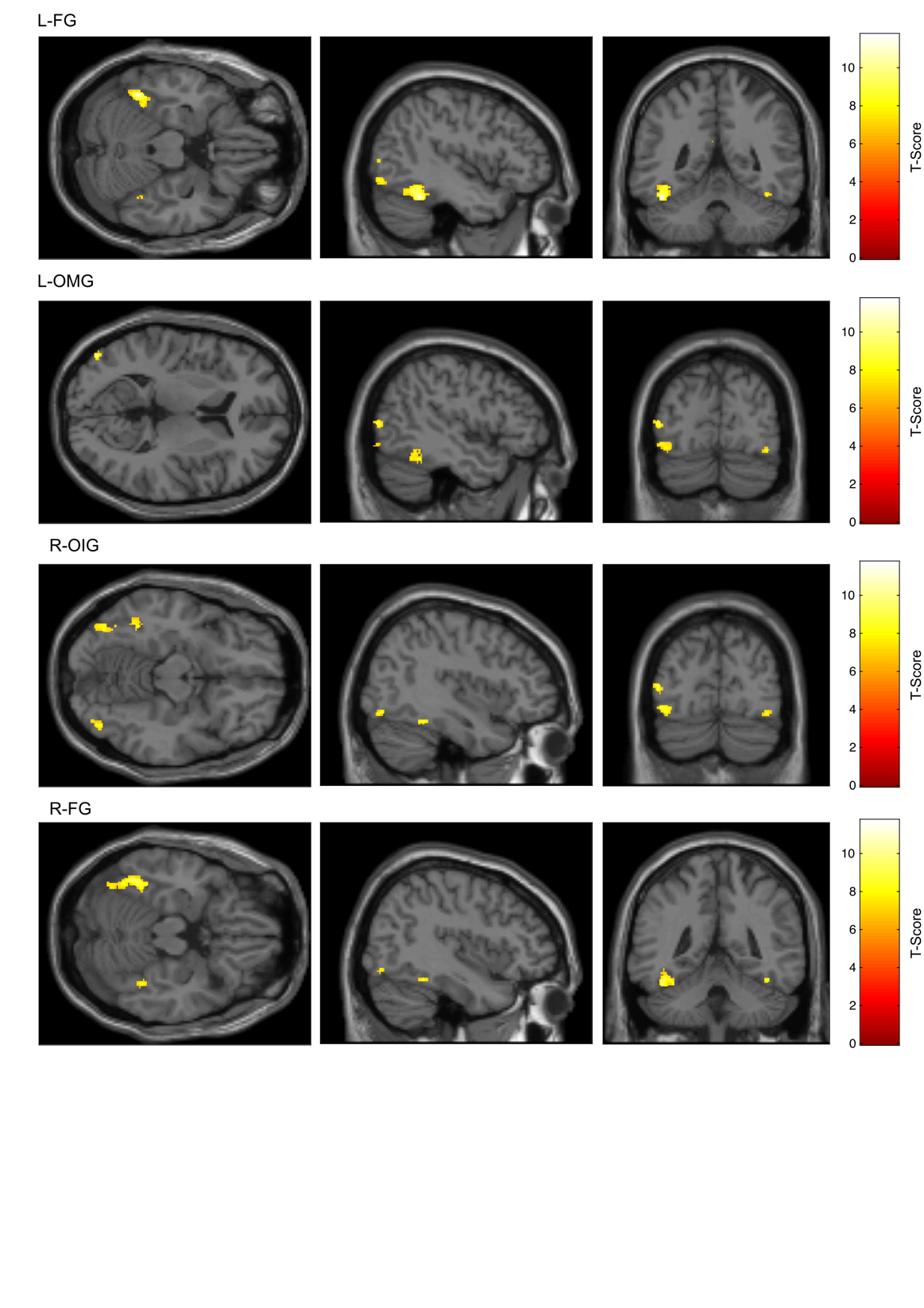


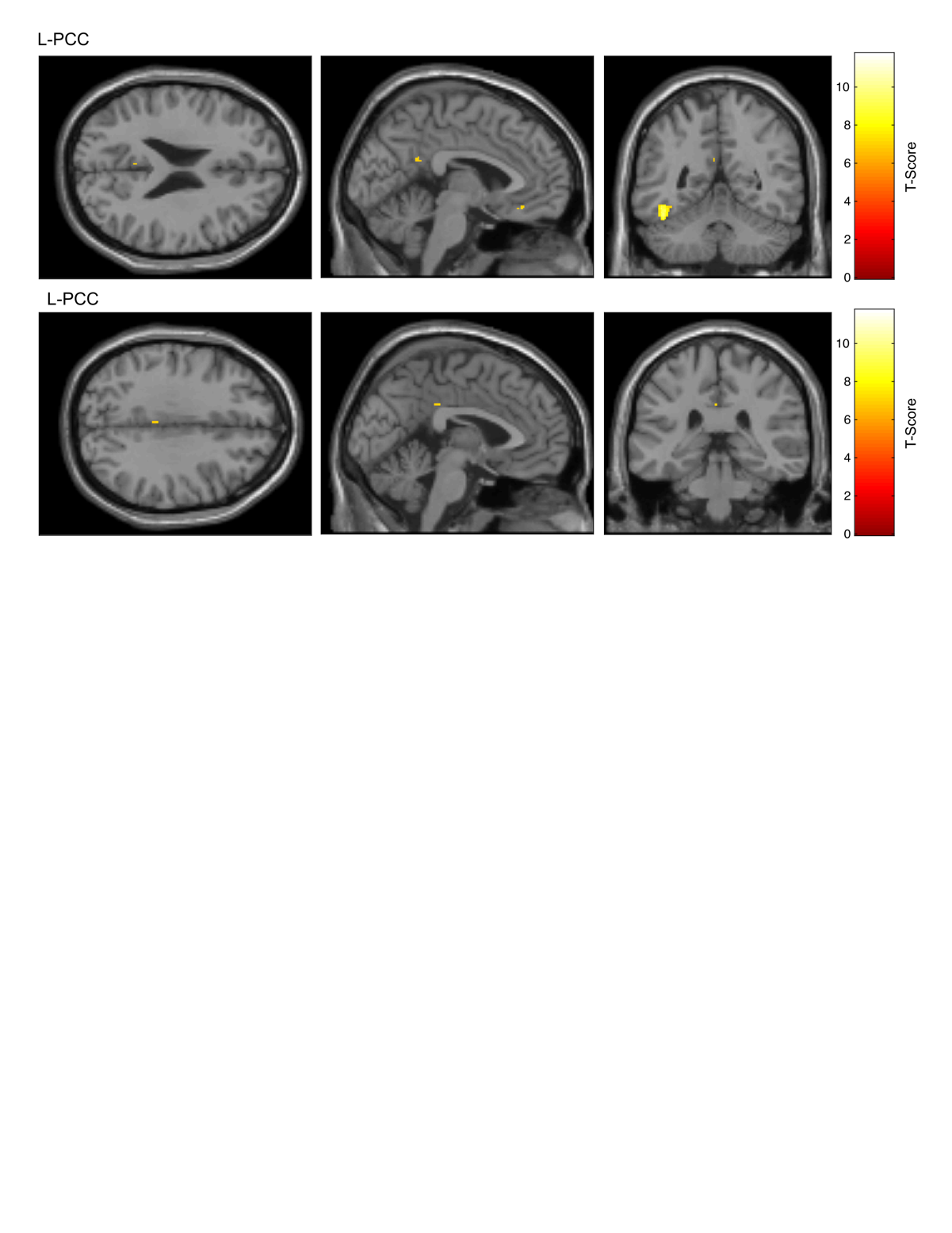


*Note*. ACR was visualized using the alcohol cue (Alc) > affectively neutral complex cue (CC) BOLD contrast (Alc-CC). The intensity and extent of activations in this contrast that survived random field theory (RFT)-based family-wise error (FWE) correction to *p*<.05 are shown on a canonical T1-weighted image of the human brain included with SPM. Data represent *N*=32 healthy emerging adults reporting regular alcohol use. L-FG=left hemisphere fusiform gyrus. L-OMG=left hemisphere occipital middle gyrus. LR-OIG=right hemisphere occipital inferior gyrus. R-FG=right hemisphere fusiform gyrus. L-PCC=left hemisphere posterior cingulate cortex.

**Figure S3.** Sex group x AUDIT subscale interaction on alcohol cue-specific BOLD reactivity in L-cmOFC


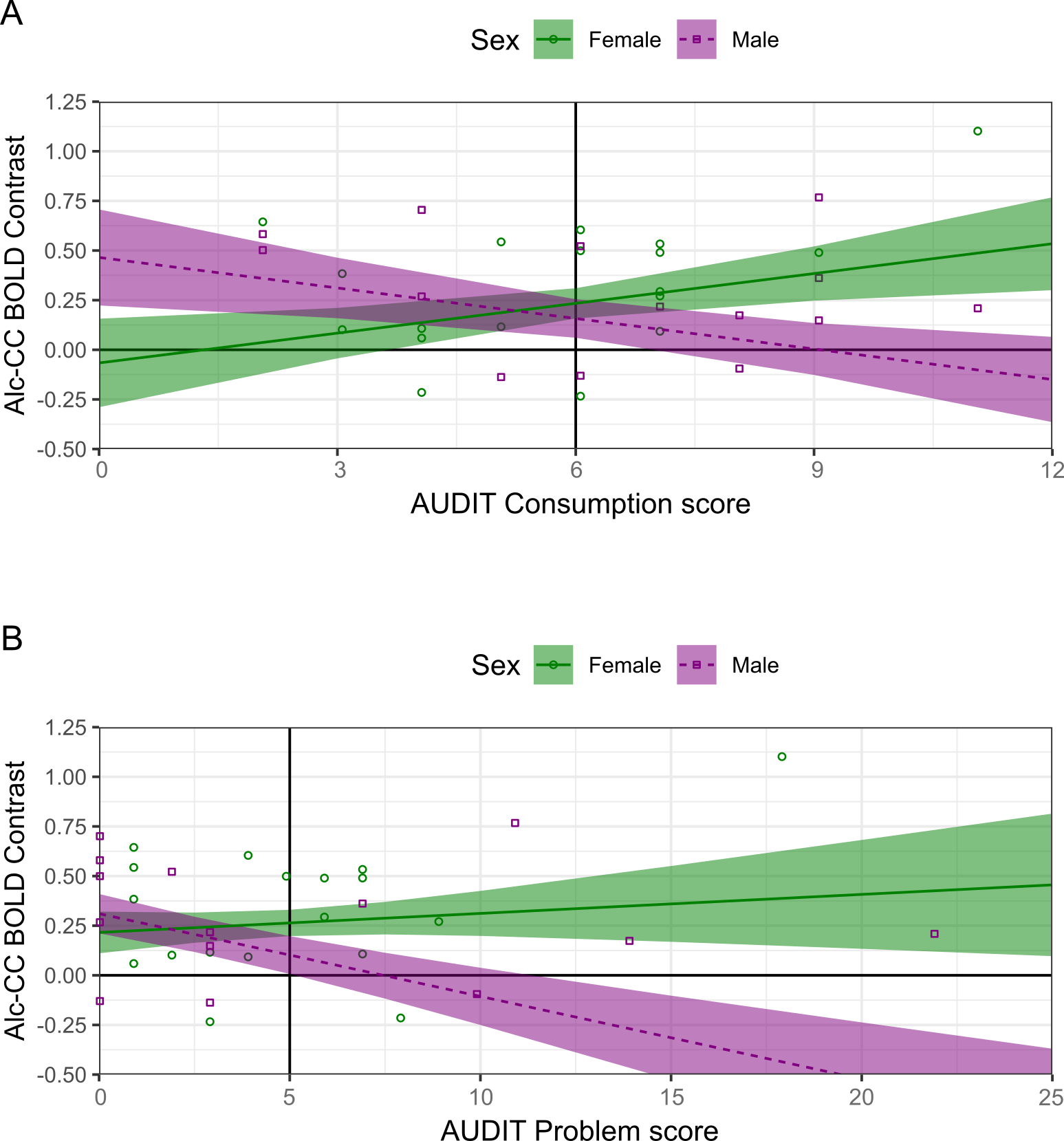


*Note*. AUDIT=Alcohol Use Disorders Identification Test. Alc=alcohol cues. CC=affectively neutral complex cues. Person-level Alc-CC BOLD contrast beta coefficients belonging to Females (*n*=18) and Males (*n*=14) are shown as circles outlined in green and purple, respectively. Multiple linear regression (MLR) model predicted M across levels of each AUDIT subscale are shown in each panel for the Females and Males as a green solid line and dashed purple line, respectively, with the boundaries of the green-filled and purple-filled areas around those lines representing ± 1 SE. AUDIT subscales were entered into the MLR models as grand-mean centered predictors. The grand-mean AUDIT subscale score is shown in each panel as a solid black vertical line intersecting the x-axis.

**Figure S4.** Sex group x Alcohol CEQ-Strength Intensity interaction on alcohol cue-specific BOLD reactivity in L-SN (**A**) and Alcohol CEQ-Frequency Intensity effect on alcohol cue-specific BOLD reactivity in R-SN (**B)**


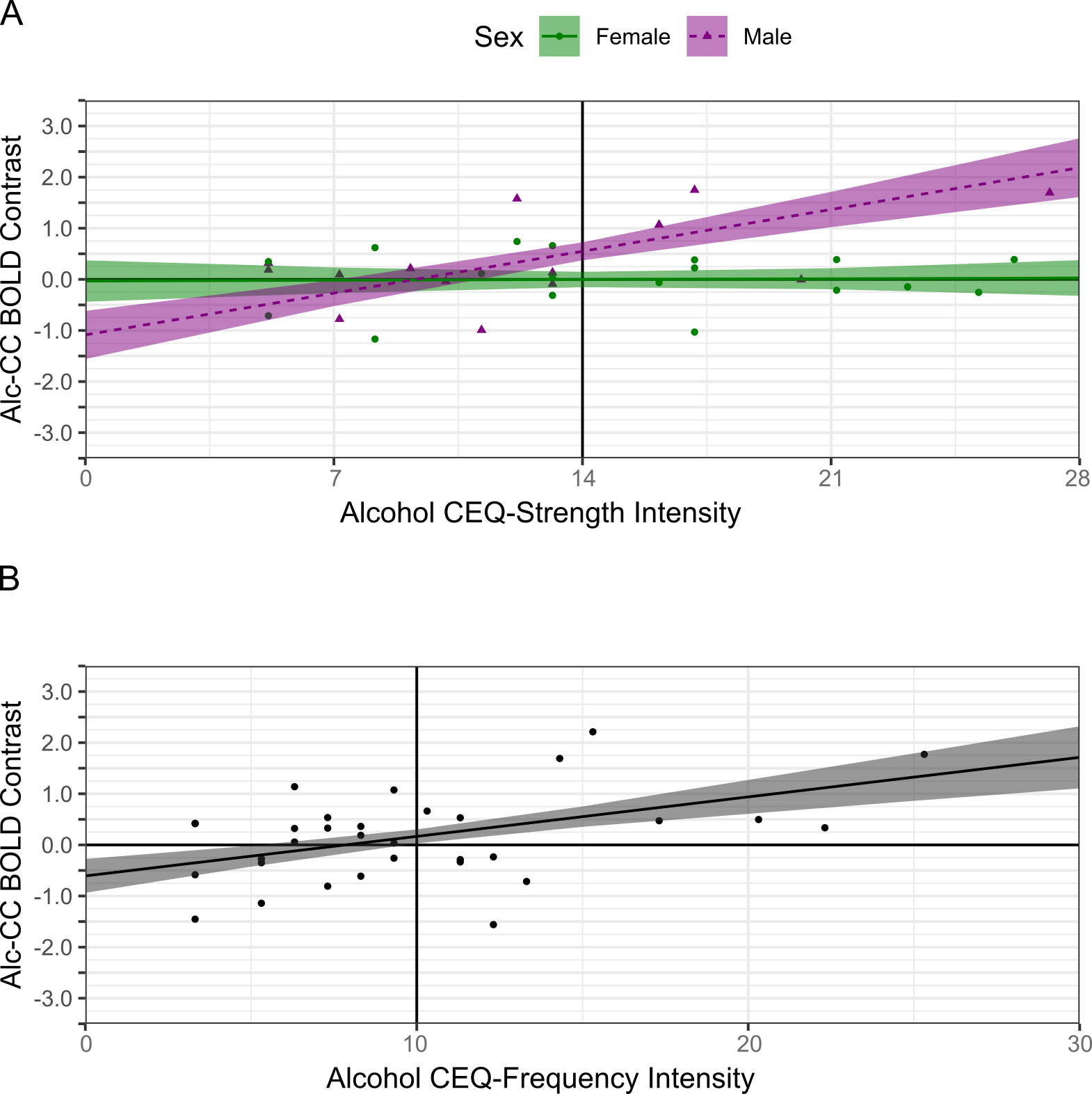


*Note*. Alc=alcohol cues. CC=affectively neutral complex cues. CEQ=Craving Experiences Questionnaire **A**: Alcohol CEQ-Strength Intensity subscale scores index the intensity level of the most intense alcohol craving experience in the past week. Person-level Alc-CC BOLD contrast beta coefficients belonging to Females (*n*=18) and Males (*n*=14) are shown as green-filled circles and purple-filled triangles, respectively. Multiple linear regression (MLR) model predicted M across levels of the Alcohol CEQ-Strength Intensity subscale are shown for the Females and Males as a green solid line and dashed purple line, respectively, with the boundaries of the green-filled and purple-filled areas around those lines representing ± 1 SE. **B:** Alcohol CEQ-Frequency Intensity subscale scores index the frequency of alcohol craving experiences in the past week. Person-level Alc-CC BOLD contrast beta coefficients are shown as black-filled circles (*N*=32). MLR model predicted M across levels of the Alcohol CEQ-Frequency Intensity subscale are shown as a black solid line with the boundaries of the gray-filled areas around the line representing ± 1 SE. **A-B:** Alcohol CEQ-Frequency and -Strength Intensity subscales were entered into the MLR models as grand-mean centered predictors. The grand-mean Alcohol CEQ-Frequency and -Strength Intensity subscale score is shown in each panel as a solid black vertical line intersecting the x-axis.

**Figure S5.** ERP responses to beverage picture cues and amplitudes of the P3/LPP component of the ERP response as a function of alcohol sensitivity phenotype group

**
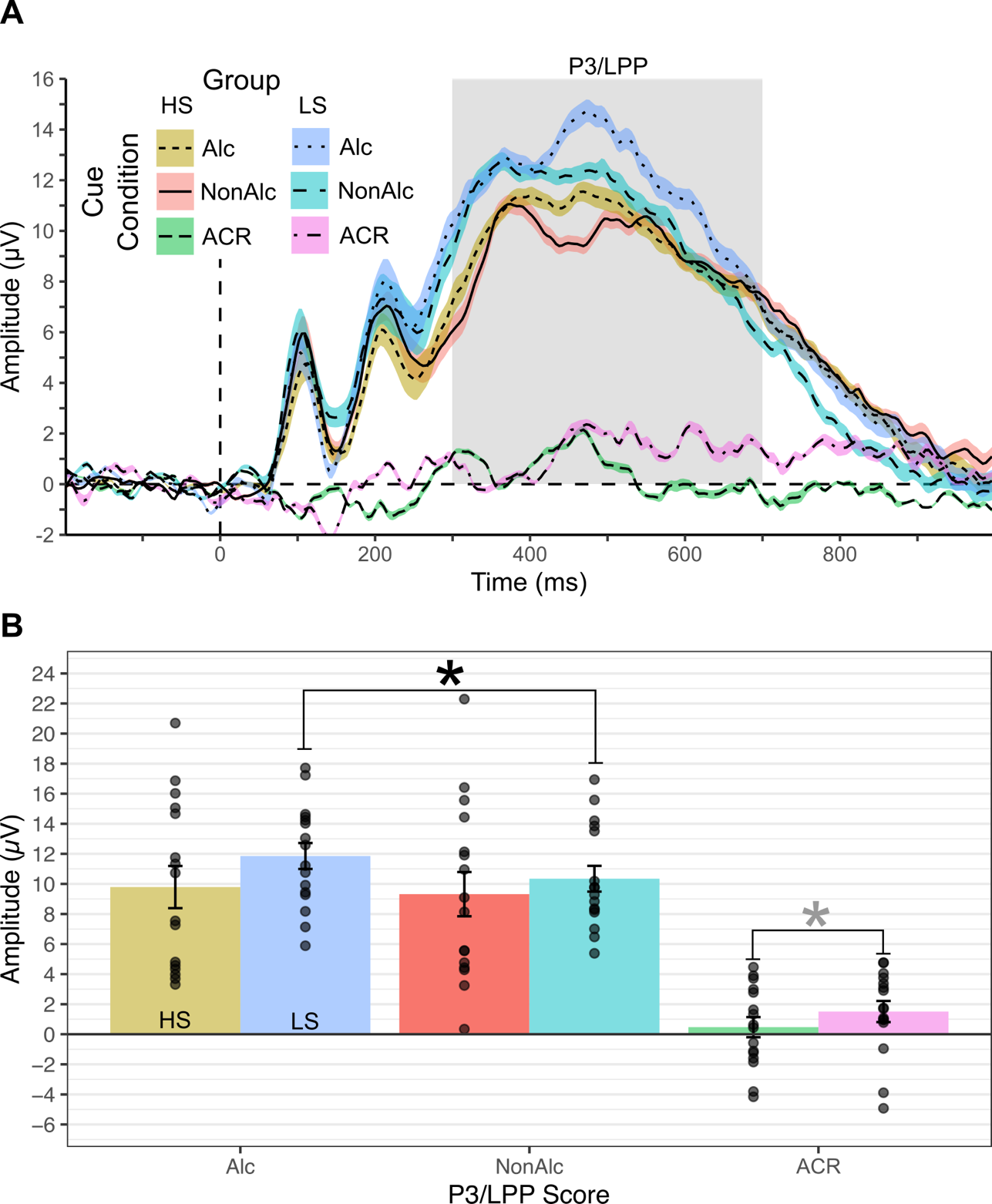
**

*Note.* **A-B**: Alc=alcohol beverage picture cues. NonAlc=non-alcohol beverage picture cues. ACR=Alc - NonAlc. **A:** ERP waveform elicited by Alc and NonAlc cues as well as the ERP difference (Alc - NonAlc) waveform for group High Sensitivity (HS, n=16) and Low Sensitivity (LS, n=16). **B:** Mean amplitude for the P3/LPP for Alc and NonAlc cues as well as the within-person difference in those mean amplitudes (i.e., ACR: Alc - NonAlc) are shown for group HS and LS. Error bars show ±1 SE. Gray-filled circles show person-level data within groups. Black asterisk=*p*<.05 for within-group within-person comparison of P3/LPP scores. Silver asterisk=*p<*.05 for between-group directional comparison (LS > HS) of within-person ACR in P3/LPP score after controlling for potential effects of sex/gender and typical alcohol use frequency across the year preceding the lab visit.

**Figure S6.** Sex group x P3/LPP ERP score interaction on alcohol cue-specific BOLD reactivity in L-vlPFC


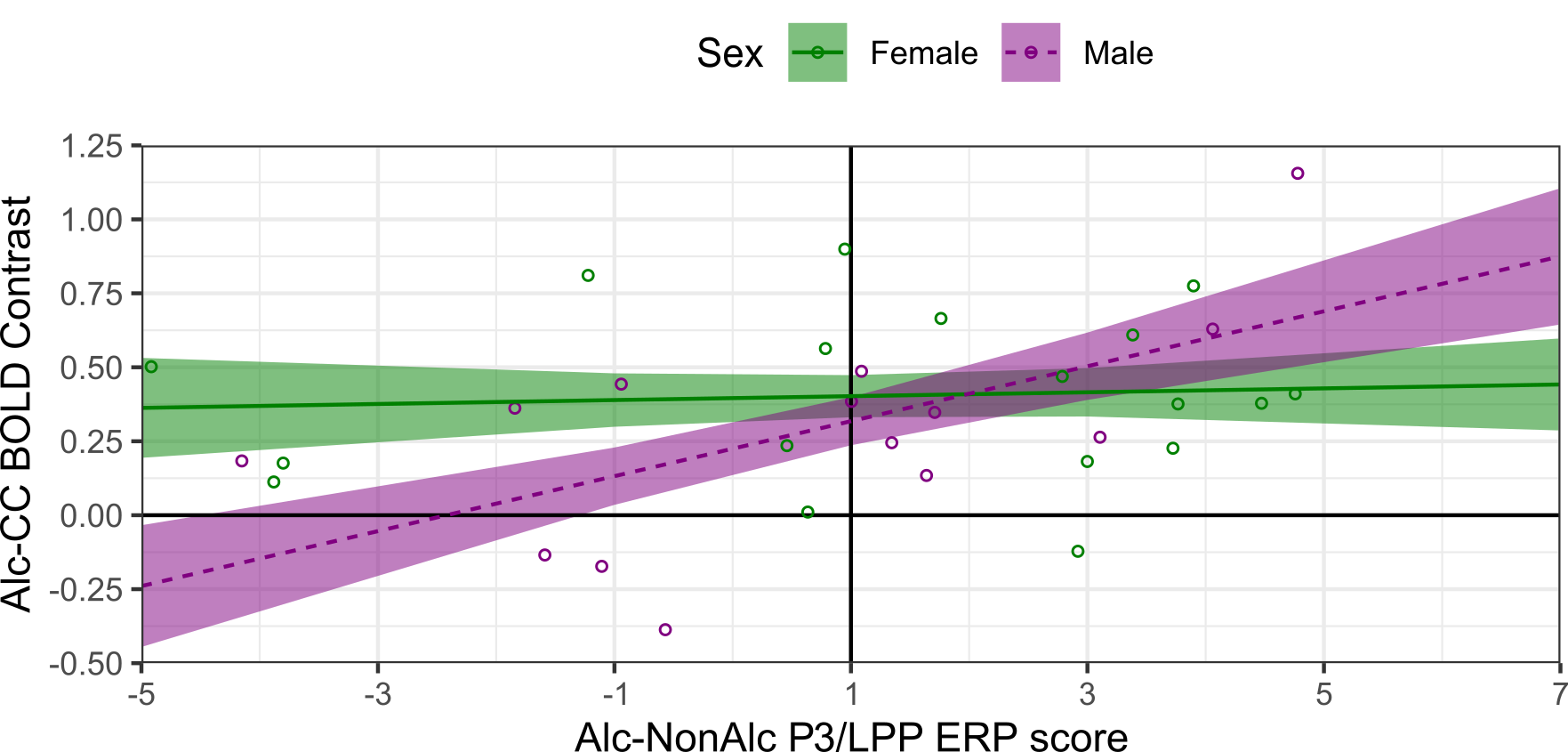


*Note*. Alc=alcohol cues. CC=affectively neutral complex cues. NonAlc=non-alcohol beverage cues. Alc-NonAlc P3/LPP ERP score is a difference in P3/LPP ERP response mean amplitude scores that indexes differential incentive salience attribution to alcohol compared to non-alcohol reward-related images. Person-level Alc-CC BOLD contrast beta coefficients belonging to Females (*n*=18) and Males (*n*=14) are shown as green-filled circles and purple-filled triangles, respectively. Multiple linear regression (MLR) model predicted M across levels of the Alc-NonAlc P3/LPP ERP score are shown for the Females and Males as a green solid line and dashed purple line, respectively, with the boundaries of the green-filled and purple-filled areas around those lines representing ± 1 SE.
